# Supplementary material for: Trends and Missing Links in (De)Hydration Research: A Narrative Review
Source: Nutrients. 2024 May 30;16(11):1709. doi: 10.3390/nu16111709 (PMC11174495; doi:10.3390/nu16111709)
Supplement: Supplementary file 1 [file nutrients-16-01709-s001.zip › OSF1 Supplement S1- Electronic Supplementary Material.pdf]

Electronic Supplementary Material

1.1. Eligibility criteria

Inclusion and exclusion criteria were based on the Population, Intervention, Comparison, Outcomes, and Study (PICOS) design:

(P) Healthy individuals of any age, ethnicity, sex, or level of physical activity. Populations comprising individuals with one or more chronic conditions were excluded. Studies comprising athletes were considered, but those with injured (e.g., studies on rehabilitation or return to sports) or disabled athletes (para-athletes) were excluded. Since the goal is to provide an overview of the research field and not provide a quantitative synthesis of outcome data, no minimum sample size was stipulated.

(I) Exposure to, or interventions focusing on, hydration strategies and dehydration (*e.g.*, induced by exercise, fluid restriction, and/or heat exposure). Studies about hydration strategies, fluid and electrolyte balance, and factors that affect hydration status and fluid balance (*e.g.*, dietary intake, exercise intensity, duration, environmental conditions, such as cool or hot environments, and types of beverages and fluids consumed) were considered. Studies where beverages supplemented with caffeine were applied were excluded.

(C) Comparators were optional and could include control groups, fully hydrated conditions, different conditions to assess the effect of dehydration on performance, identification of types of beverages and fluids consumed by participants, and evaluation of the effectiveness of hydration strategies in maintaining fluid balance and blood sodium concentration.

(O) The considered outcomes for inclusion comprised hydration status, fluid balance, electrolyte balance, types and amounts of beverages consumed by athletes during competitive sporting activities, ergometry performance, performance, or health outcomes.

(S) Any interventional or observational study, either with a cross-sectional or longitudinal design. Commentaries, editorials, letters, and meeting abstracts were excluded.

1.2. Information sources and search strategy

Searches were conducted across three databases (PubMed, EMBASE, and Web of Science) from their inception until 9 May 2023 (after protocol registration). The search used both specific Medical Subject Headings (MeSH terms) and keywords in natural language: (i) "hydration" OR "dehydration" OR "hypohydration" AND (ii) "sports drink*" OR "beverag*". The entire search strategy is presented in Supplementary Table **1**. Only articles written in English were accepted. Manual searches were performed within the reference lists of the eligible studies for inclusion and representative systematic reviews on hydration/dehydration.

Supplementary Table 1. Resulting code lines for each database

Database	Specificities of each database	Code lines generated by the database after performing the searches
PubMed	Nothing to report	(("hydration"[Title/Abstract] OR "dehydration"[Title/Abstract] OR "hypohydration"[Title/Abstract]) AND "sports drink*"[Title/Abstract]) OR "beverag*"[Title/Abstract])
EMBASE	Nothing to report	(('hydration':ti,ab OR 'dehydration':ti,ab OR 'hypohydration':ti,ab) AND 'sports drink*':ti,ab) OR 'beverag*':ti,ab
Web of Science	Title, abstract and keywords (termed “Topic”)	(TS=("hydration" OR "dehydration" OR "hypohydration")) AND TS=("sports drink*" OR "beverage*")

1.3. Data Collection

Studies were screened to collect and systematize data regarding all essential aspects of the literature relevant to this review, including author, year, country, aim(s), study design, sample size, sex, age, competitive level, environmental conditions, beverage characteristics, (de)hydration strategy, and primary outcomes. The complete list of data extracted is detailed below.

(i) Publication-related information:

a. Publication date
b. Country of origin
c. Study design: observation or interventional studies. Within the interventional, randomized, or non-randomized study

(ii) Participant-related information:

a. Sample size, age, sex
b. The competitive level was reported according to the Participant Classification Framework [1].

(iii) Strategy-based evidence-level:

a. A color coding was used to denote observational studies (pink) and interventional studies applying different beverages (green), similar beverages but different compositions (orange), similar beverages at different temperatures (blue), similar beverages at different intake kinetics (yellow), other (gray), or a dehydration protocol. One study may be included in more than one category. Studies, where fluids were taken at different rates, were considered studies analyzing the ingestion of fluids at different intake kinetics.

(iv) Exercise protocol information:

a. Exercise type and protocol length (e.g., duration in min, until exhaustion, % body weight lost)

b. Environmental conditions (e.g., temperature, relative humidity, wind speed)

(v) Intake protocol information:

a. Beverage type (e.g., sports drink, CHO-E, E (electrolytes), CHO (carbohydrate), water, Gatorade, Powerade, milk, ORS (oral rehydration solution), CHO-AA, CHO-PRO (proteins), E-AA (amino acids), AA, energy drink, others, unknown placebo, glycerol beverage, CHO-E-AA.
b. Beverage composition (e.g., magnesium, potassium, calcium, sodium, water, chloride, AA, protein, CHO, sugars, and others)
c. Intake strategy (e.g., *ad libitum*, concrete volume fluid, % body weight loss)
d. Within-session timing (e.g., pre-set, inter-set, post-set). Herein set is meant for exercise. A color coding was used to denote studies applying an inter-set intake strategy (green), a post-set strategy (blue), a pre-set strategy (yellow), a pre-set and inter-set strategy (orange), a pre-set and post-set strategy (brown), an inter-set and post-set strategy (gray), a pre-set, inter-set, and post-set strategy (dark green), and for studies with non-applicable strategy (dark blue).
e. Within-season timing (e.g., pre-season, competitive season, post-season) exclusively for studies comprising a cohort of athletes.

(vi) Outcomes information:

a. Outcome domain (a color coding was utilized to denote studies with hydration status outcomes (purple), performance outcomes (blue), health outcomes (green), hydration and performance outcomes (pink), hydration and health outcomes (gray), performance and health outcomes (orange) and hydration, performance, and health outcomes (yellow))
b. Performance outcomes (e.g., total work, performance time, power output)
c. Health outcomes (e.g., heart rate, blood glucose, subjective feelings)

d. Hydration outcomes (e.g., urine color, urine Na^+ concentration, plasma osmolality)

1.4. Data management and synthesis

We perform a descriptive summary of study characteristics and then a thematic synthesis related to (de)hydration. The participant's physical activity level was characterized according to the Participation Classification Framework (PCF) [1], using the classification reported in the study or, if unavailable, the classification attributed by the authors. Subjects that fulfilled the Tier 0 or 1 requirements of the PCF [1] were classified as non-athletes. Those who fulfilled the criteria for at least Tier 2 of the PCF [1] were entitled as athletes. The type of outcomes was collected for summary purposes, but not their numerical results. To provide an overview of the existing body and the corresponding gaps in research, an EGM was constructed to graphically represent the body of evidence and intuitively convey an overview of the existing evidence and the current research gaps [2-4]. In the EGM, the different circles have proportional sizes, reflecting the number of studies; however, this proportionality is only applied within each cell and not between cells. More detailed information on data management and synthesis on each topic is described below.

Participant-related information

Despite “gender” being generally used in English, this is a psychosocial construct of complex evaluation. Yet sex is in the simplest biological sense, and so is the terminology we adopted in this review [5, 6]. Studies that failed to detail sex were described as unreported, potentially meaning they were also males, contemplating societal bias. Two studies [7, 8] did not report the number of included females and males, despite reporting the inclusion of both sexes.

For age information, we presented it as the mean/median (rounded to one decimal place when feasible, unless the authors exclusively provided whole units without decimal places), or if this data was unavailable, we indicated the age range. Our primary focus was to present the age of the entire sample. Age-related details for specific

groups within a study were only included if the authors did not provide an overall age summary. In those cases, a mean of the ages of those groups was calculated and considered. The age pyramid was constructed using the mean of the ages and based on the National Institutes of Health data [9]. Only one study [10], mentioned subjects older than 65 years.

Exercise protocol

Concerning exercise length, the duration of exercise was standardized to minutes despite the unit used in primary studies. To avoid subjectiveness on our results, not all studies were possibly included in this analysis since relevant information (such as exercise duration) was not reported. Only the studies that clearly described the time of the exercise protocol were considered in this review analysis. Hence, of 180 studies, 143 were included in this analysis.

Regarding environmental conditions, only temperature and relative humidity were used in this review, considering that those were the two most cited ecological parameters in the included studies. Based on World Health Organization (WHO) guidelines [11], environments with temperatures ranging from 8 to 15 °C were defined as relaxed environments, while if the temperature was between 15 to 30 °C, they were considered environments at room temperature (RT). Additionally, environments with temperatures above 30 °C were classified as hot environments. Dry bulb temperature was always considered over wet bulb temperature. Most people rate a relative humidity between 30 to 60% as the most comfortable, regardless of the ideal indoor humidity being from 30 to 50% [12]. Hence, atmospheres with humidity below 30% or above 60% were considered below or above the normal, respectively. Atmospheres with relative humidity ranging between the two stated values were taken as normal. Conditions COOL/RT and HOT/RT result from studies where two interventions were performed under different environmental temperatures or from studies where temperature ranged between two conditions.

Intake protocol

Pertaining intake strategy, the concrete volume of fluid refers to the total volume ingested during the protocol. Such volume was calculated multiplying the quantity of

fluid taken each interval of time (when clearly described in the article) by the length of the exercise protocol (when clearly described in the article). To avoid subjectiveness on our results, not all studies were possible of being included in this analysis since relevant information (such as number of intakes being dependent on exercise duration) was not clear reported. Hence, of 180 studies, 140 were included in this analysis. All results consider all fluids taken either pre, during, or after exercise (no distinction between these moments was made). 1 L was considered equivalent to 1 kg.

No distinction was made between control beverages and study beverages since most studies were not clear regarding this information.

Outcomes

Performance outcomes were encoded into 22 different parameters: muscle free glucose, expired gas 13C:12C ratio, respiratory outputs, VCo2 (volume of carbon dioxide breathe out), substrate utilization, VO_2 (oxygen consumption), PO/VO_2 ratio, PCr (phosphocreatine), lactic acid concentrations, felt arousal scale, muscle damage, energy expenditure, creatine kinase levels, myoglobin concentration, muscle glycogen concentration, torque, PO (power output), total work, performance skill, time to exhaustion, performance time, and RPE (rate of perceived exertion).

Health outcomes were clustered into 16 different parameters: physiological strain index, metabolic heat production/storage, thermal comfort, subjective feelings, taste perceptions, DH2 enrichment, blood cells count, temperature, hematocrit/hemoglobin concentrations, vital signs, blood pH, mental fatigue, cognitive capacity, glycemic response, blood measurements, and heart rate.

Finally hydration outcomes were prearranged into 46 different parameters: urinary 3MH-I (3-methylhistidine), gastric osmolality, gastric electrolytes, urine inulin, urine osmolality/plasma osmolality ratio, urine pH, plasma osmotic pressure, sweat osmolality, saliva outputs, non-renal water losses, plasma ADH (antidiuretic hormone), respiratory losses, change in renal Na^+ absorption, renin activity, gastric outputs, estimated glomerular filtration rate, net fluid balance, BHI (beverage hydration index), sweat outputs, USG (urine specific gravity), extracellular water, intracellular water, total body water, free water reserve, plasma aldosterone, plasma electrolytes, saliva osmolality, plasma osmolality, sweat electrolytes, urine electrolytes, urine osmolality,

urine antioxidant properties, blood urena nitrogen, urine creatinine excretion, urine color, urine outputs, cell volume, rehydration %; metabolic loss, time do dehydration (3% body mass loss), dehydration %, plasma volume changes, body weight changes, fluid loss, fluid retention, fluid intake.

1.5. Selection of studies for inclusion

The initial search identified 78,873 studies. After duplicate removal (n= 32,187), 46,686 unique records remained for title and abstract screening. Further analysis excluded 46,188 studies. A total of 498 studies underwent full-text screening. Six studies were not available online and/or corresponding authors could not provide the studies or did not reply to the authors' request. Exclusion criteria determined a further removal of 312 studies due to inappropriate theme (not about hydration), study design, study cohort (not individuals or healthy individuals), and/or language (not written in English). Hand searches yielded 249 records, all of which were duplicated from database searches. A total of 180 studies [7, 8, 10, 13-188] fulfilled the eligibility criteria and were included in this review (Supplementary Figure 1).

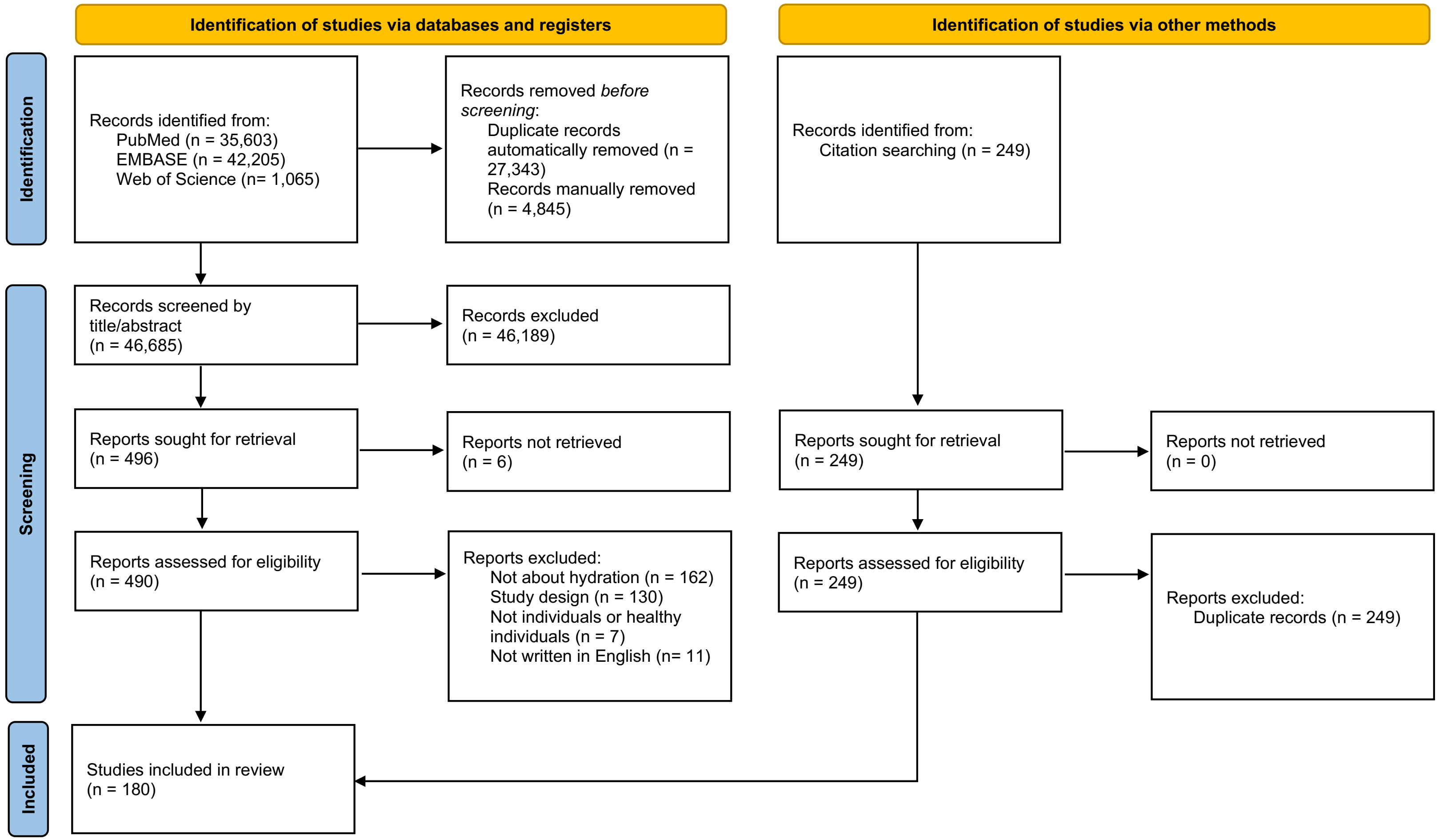

Supplementary Figure 1. PRISMA 2020 flow diagram.

1.6. Further supplementary material

The description of the full information for each study included in the analysis (data extracted related to publication and participant-level details, interventional-level details, and beverages and outcomes details) is available in the OSF database: **https://osf.io/5q9xt**.

The list of files available in the dataset is as follows:

- Supplementary OSF 1: Electronic Supplementary Material
- Supplementary OSF 2: Publication and Participant-level details
- Supplementary OSF 3: Intervention-level details
- Supplementary OSF 4: Beverages and Outcomes details

References

1. McKay, A.K.A., et al., *Defining Training and Performance Caliber: A Participant Classification Framework.* Int J Sports Physiol Perform, 2022. **17**(2): p. 317-331.
2. Miake-Lye, I.M., et al., *What is an evidence map? A systematic review of published evidence maps and their definitions, methods, and products.* Syst Rev, 2016. **5**: p. 28.
3. Schuller-Martinez, B., et al., *Graphical representation of the body of the evidence: the essentials for understanding the evidence gap map approach.* Medwave, 2021. **21**(3): p. e8164.
4. Snilstveit, B., et al., *Evidence & Gap Maps: A tool for promoting evidence informed policy and strategic research agendas.* J Clin Epidemiol, 2016. **79**: p. 120-129.
5. Gogovor, A., et al., *Sex and gender considerations in reporting guidelines for health research: a systematic review.* Biol Sex Differ, 2021. **12**(1): p. 62.
6. Clayton, J.A. and C. Tannenbaum, *Reporting Sex, Gender, or Both in Clinical Research?* JAMA, 2016. **316**(18): p. 1863-1864.
7. Mora-Rodriguez, R., et al., *Influence of Physical Activity and Ambient Temperature on Hydration: The European Hydration Research Study (EHRS).* Nutrients, 2016. **8**(5).
8. Utter, A.C., et al., *Effect of carbohydrate ingestion on ratings of perceived exertion during a marathon.* Med Sci Sports Exerc, 2002. **34**(11): p. 1779-84.
9. Health, N.I.o., *Age.* 2023.
10. Clarke, M.M., et al., *A randomized trial to assess beverage hydration index in healthy older adults.* The American journal of clinical nutrition, 2019. **109**(6): p. 1640-1647.
11. Foundation, E. *"Ambient", "Room Temperature", "Cold " - what is what?* 2023 September 09, 2018 [cited 2023; Available from: https://www.gmp-compliance.org/gmp-news/ambient-room-temperature-cold-what-is-what.
12. Australia, N.A.C. *Indoor humidity and your family's health*. 2023 [cited November 2023; Available from: https://www.nationalasthma.org.au/news/2016/indoor-humidity.
13. Owen, M.D., et al., *Effects of ingesting carbohydrate beverages during exercise in the heat.* Med Sci Sports Exerc, 1986. **18**(5): p. 568-75.
14. Davis, J.M., et al., *Effects of ingesting 6% and 12% glucose/electrolyte beverages during prolonged intermittent cycling in the heat.* Eur J Appl Physiol Occup Physiol, 1988. **57**(5): p. 563-9.

15. Davis, J.M., et al., *Carbohydrate-electrolyte drinks: effects on endurance cycling in the heat.* Am J Clin Nutr, 1988. **48**(4): p. 1023-30.
16. Carter, J.E. and C.V. Gisolfi, *Fluid replacement during and after exercise in the heat.* Med Sci Sports Exerc, 1989. **21**(5): p. 532-9.
17. Murray, R., et al., *Carbohydrate feeding and exercise: effect of beverage carbohydrate content.* Eur J Appl Physiol Occup Physiol, 1989. **59**(1-2): p. 152-8.
18. Davis, J.M., et al., *Fluid availability of sports drinks differing in carbohydrate type and concentration.* Am J Clin Nutr, 1990. **51**(6): p. 1054-7.
19. Lyons, T.P., et al., *Effects of glycerol-induced hyperhydration prior to exercise in the heat on sweating and core temperature.* Med Sci Sports Exerc, 1990. **22**(4): p. 477-83.
20. Powers, S.K., et al., *Fluid replacement drinks during high intensity exercise: effects on minimizing exercise-induced disturbances in homeostasis.* Eur J Appl Physiol Occup Physiol, 1990. **60**(1): p. 54-60.
21. Barr, S.I., D.L. Costill, and W.J. Fink, *Fluid replacement during prolonged exercise: effects of water, saline, or no fluid.* Med Sci Sports Exerc, 1991. **23**(7): p. 811-7.
22. Ryan, A.J., A.E. Navarre, and C.V. Gisolfi, *Consumption of carbonated and noncarbonated sports drinks during prolonged treadmill exercise in the heat.* Int J Sport Nutr, 1991. **1**(3): p. 225-39.
23. Seidman, D.S., et al., *The effects of glucose polymer beverage ingestion during prolonged outdoor exercise in the heat.* Med Sci Sports Exerc, 1991. **23**(4): p. 458-62.
24. Criswell, D., et al., *Fluid replacement beverages and maintenance of plasma volume during exercise: role of aldosterone and vasopressin.* Eur J Appl Physiol Occup Physiol, 1992. **65**(5): p. 445-51.
25. Lambert, C.P., et al., *Fluid replacement after dehydration: influence of beverage carbonation and carbohydrate content.* Int J Sports Med, 1992. **13**(4): p. 285-92.
26. Millard-Stafford, M.L., et al., *Carbohydrate-electrolyte replacement improves distance running performance in the heat.* Med Sci Sports Exerc, 1992. **24**(8): p. 934-40.
27. Widrick, J.J., et al., *Carbohydrate feedings and exercise performance: effect of initial muscle glycogen concentration.* J Appl Physiol (1985), 1993. **74**(6): p. 2998-3005.
28. Meyer, F., et al., *Hypohydration during exercise in children: effect on thirst, drink preferences, and rehydration.* Int J Sport Nutr, 1994. **4**(1): p. 22-35.
29. Hickey, M.S., D.L. Costill, and S.W. Trappe, *Drinking behavior and exercise-thermal stress: role of drink carbonation.* Int J Sport Nutr, 1994. **4**(1): p. 8-21.
30. Maughan, R.J., et al., *Post-exercise rehydration in man: effects of electrolyte addition to ingested fluids.* Eur J Appl Physiol Occup Physiol, 1994. **69**(3): p. 209-15.
31. Millard-Stafford, M., et al., *Fluid intake in male and female runners during a 40-km field run in the heat.* J Sports Sci, 1995. **13**(3): p. 257-63.
32. Glickman-Weiss, E.L., et al., *A comparison of a carbohydrate–electrolyte beverage versus a placebo beverage in maintaining thermoregulatory and blood homeostasis during the training of fire fighters.* Wilderness & Environmental Medicine, 1995. **6**(4): p. 377-384.
33. Wilk, B. and O. Bar-Or, *Effect of drink flavor and NaCL on voluntary drinking and hydration in boys exercising in the heat.* J Appl Physiol (1985), 1996. **80**(4): p. 1112-7.
34. Febbraio, M.A., et al., *Effect of CHO ingestion on exercise metabolism and performance in different ambient temperatures.* Med Sci Sports Exerc, 1996. **28**(11): p. 1380-7.
35. el-Sayed, M.S., J. Balmer, and A.J. Rattu, *Carbohydrate ingestion improves endurance performance during a 1 h simulated cycling time trial.* J Sports Sci, 1997. **15**(2): p. 223-30.
36. Davis, J.M., et al., *Carbohydrate drinks delay fatigue during intermittent, high-intensity cycling in active men and women.* Int J Sport Nutr, 1997. **7**(4): p. 261-73.
37. Koulmann, N., et al., *Effects of different carbohydrate-electrolyte beverages on the appearance of ingested deuterium in body fluids during moderate exercise by humans in the heat.* Eur J Appl Physiol Occup Physiol, 1997. **75**(6): p. 525-31.

38. Millard-Stafford, M., et al., *Water versus carbohydrate-electrolyte ingestion before and during a 15-km run in the heat.* Int J Sport Nutr, 1997. **7**(1): p. 26-38.
39. Wong, S.H., et al., *Influence of fluid intake pattern on short-term recovery from prolonged, submaximal running and subsequent exercise capacity.* J Sports Sci, 1998. **16**(2): p. 143-52.
40. Wilk, B., et al., *Consistency in preventing voluntary dehydration in boys who drink a flavored carbohydrate-NaCl beverage during exercise in the heat.* Int J Sport Nutr, 1998. **8**(1): p. 1-9.
41. Palmer, G.S., et al., *Carbohydrate ingestion immediately before exercise does not improve 20 km time trial performance in well trained cyclists.* Int J Sports Med, 1998. **19**(6): p. 415-8.
42. Rivera-Brown, A.M., et al., *Drink composition, voluntary drinking, and fluid balance in exercising, trained, heat-acclimatized boys.* J Appl Physiol (1985), 1999. **86**(1): p. 78-84.
43. Vrijens, D.M. and N.J. Rehrer, *Sodium-free fluid ingestion decreases plasma sodium during exercise in the heat.* J Appl Physiol (1985), 1999. **86**(6): p. 1847-51.
44. Davis, J.M., et al., *Effects of branched-chain amino acids and carbohydrate on fatigue during intermittent, high-intensity running.* Int J Sports Med, 1999. **20**(5): p. 309-14.
45. Sanders, B., T.D. Noakes, and S.C. Dennis, *Water and electrolyte shifts with partial fluid replacement during exercise.* Eur J Appl Physiol Occup Physiol, 1999. **80**(4): p. 318-23.
46. Utter, A.C., et al., *Effect of carbohydrate ingestion and hormonal responses on ratings of perceived exertion during prolonged cycling and running.* Eur J Appl Physiol Occup Physiol, 1999. **80**(2): p. 92-9.
47. Mitchell, J.B., et al., *Postexercise rehydration: effect of Na(+) and volume on restoration of fluid spaces and cardiovascular function.* J Appl Physiol (1985), 2000. **89**(4): p. 1302-9.
48. Clapp, A.J., et al., *Effects of carbohydrate-electrolyte content of beverages on voluntary hydration in a simulated industrial environment.* AIHAJ, 2000. **61**(5): p. 692-9.
49. Mitchell, J., et al., *Pre-exercise carbohydrate and fluid ingestion: influence of glycemic response on 10-km treadmill running performance in the heat.* Journal of sports medicine and physical fitness, 2000. **40**(1): p. 41.
50. Febbraio, M.A., et al., *Effects of carbohydrate ingestion before and during exercise on glucose kinetics and performance.* J Appl Physiol (1985), 2000. **89**(6): p. 2220-6.
51. Warber, J.P., et al., *The effects of choline supplementation on physical performance.* Int J Sport Nutr Exerc Metab, 2000. **10**(2): p. 170-81.
52. Niles, E.S., et al., *Carbohydrate-protein drink improves time to exhaustion after recovery from endurance exercise.* Journal of Exercise Physiology Online, 2001. **4**(1).
53. Wojcik, J.R., et al., *Comparison of carbohydrate and milk-based beverages on muscle damage and glycogen following exercise.* Int J Sport Nutr Exerc Metab, 2001. **11**(4): p. 406-19.
54. Horie, S., T. Tsutsui, and S. Miyazaki, *Effect of dilution of sports drink on water balance and beverage preference of heat-exposed steel workers.* Journal of UOEH, 2003. **25**(1): p. 1-11.
55. Aoki, M.S., et al., *Carbohydrate supplementation fails to revert the deleterious effects of endurance exercise upon subsequent strength performance.* Revista Brasileira de Medicina do Esporte, 2003. **9**: p. 282-287.
56. Williams, M.B., et al., *Effects of recovery beverages on glycogen restoration and endurance exercise performance.* The Journal of Strength & Conditioning Research, 2003. **17**(1): p. 12-19.
57. Saunders, M.J., M.D. Kane, and M.K. Todd, *Effects of a carbohydrate-protein beverage on cycling endurance and muscle damage.* Med Sci Sports Exerc, 2004. **36**(7): p. 1233-8.

58. Wingo, J.E., et al., *Influence of a Pre-Exercise Glycerol Hydration Beverage on Performance and Physiologic Function During Mountain-Bike Races in the Heat.* J Athl Train, 2004. **39**(2): p. 169-175.
59. FINN, K.J., F.A. DOLGENER, and R.B. WILLIAMS, *EFFECTS OF CARBOHYDRATE REFEEDING ON PHYSIOLOGICAL RESPONSES AND PSYCHOLOGICAL AND PHYSICAL PERFORMANCE FOLLOWING ACUTE WEIGHT REDUCTION IN COLLEGIATE WRESTLERS.* The Journal of Strength & Conditioning Research, 2004. **18**(2): p. 328-333.
60. Utter, A.C., et al., *Carbohydrate supplementation and perceived exertion during prolonged running.* Med Sci Sports Exerc, 2004. **36**(6): p. 1036-41.
61. Millard-Stafford, M.L., et al., *Should carbohydrate concentration of a sports drink be less than 8% during exercise in the heat?* Int J Sport Nutr Exerc Metab, 2005. **15**(2): p. 117-30.
62. Backhouse, S.H., et al., *Effect of carbohydrate and prolonged exercise on affect and perceived exertion.* Med Sci Sports Exerc, 2005. **37**(10): p. 1768-73.
63. Utter, A.C., et al., *Carbohydrate supplementation and perceived exertion during resistance exercise.* The Journal of Strength & Conditioning Research, 2005. **19**(4): p. 939-943.
64. de Carvalho, M.V., J.C. Marins, and E. Silami-Garcia, *The influence of water versus carbohydrate-electrolyte hydration on blood components during a 16-km military march.* Mil Med, 2007. **172**(1): p. 79-82.
65. Ismail, I., R. Singh, and R. Sirisinghe, *Rehydration with sodium-enriched coconut water after exercise-induced dehydration.* Southeast Asian journal of tropical medicine and public health, 2007. **38**(4): p. 769.
66. Maughan, R.J., et al., *Water balance and salt losses in competitive football.* Int J Sport Nutr Exerc Metab, 2007. **17**(6): p. 583-94.
67. Shirreffs, S.M., P. Watson, and R.J. Maughan, *Milk as an effective post-exercise rehydration drink.* Br J Nutr, 2007. **98**(1): p. 173-80.
68. Wilk, B., A.M. Rivera-Brown, and O. Bar-Or, *Voluntary drinking and hydration in non-acclimatized girls exercising in the heat.* Eur J Appl Physiol, 2007. **101**(6): p. 727-34.
69. Baty, J.J., et al., *The effect of a carbohydrate and protein supplement on resistance exercise performance, hormonal response, and muscle damage.* The Journal of Strength & Conditioning Research, 2007. **21**(2): p. 321-329.
70. Shirreffs, S.M., et al., *Rehydration after exercise in the heat: a comparison of 4 commonly used drinks.* International journal of sport nutrition and exercise metabolism, 2007. **17**(3): p. 244-258.
71. Luden, N.D., M.J. Saunders, and M.K. Todd, *Postexercise carbohydrate-protein-antioxidant ingestion decreases plasma creatine kinase and muscle soreness.* Int J Sport Nutr Exerc Metab, 2007. **17**(1): p. 109-23.
72. Goulet, E.D., et al., *Pre-exercise hyperhydration delays dehydration and improves endurance capacity during 2 h of cycling in a temperate climate.* J Physiol Anthropol, 2008. **27**(5): p. 263-71.
73. Hill, R.J., L.J. Bluck, and P.S. Davies, *The hydration ability of three commercially available sports drinks and water.* J Sci Med Sport, 2008. **11**(2): p. 116-23.
74. Rivera-Brown, A.M., et al., *Voluntary drinking and hydration in trained, heat-acclimatized girls exercising in a hot and humid climate.* Eur J Appl Physiol, 2008. **103**(1): p. 109-16.
75. Sun, J.M., et al., *Dehydration rates and rehydration efficacy of water and sports drink during one hour of moderate intensity exercise in well-trained flatwater kayakers.* Annals Academy of Medicine Singapore, 2008. **37**(4): p. 261.
76. Bailey, S.P., et al., *Impact of prolonged exercise in the heat and carbohydrate supplementation on performance of a virtual environment task.* Mil Med, 2008. **173**(2): p. 187-92.

77. Currell, K. and A.E. Jeukendrup, *Superior endurance performance with ingestion of multiple transportable carbohydrates.* Med Sci Sports Exerc, 2008. **40**(2): p. 275-81.
78. Davison, G.W., et al., *The effects of ingesting a carbohydrate-electrolyte beverage 15 minutes prior to high-intensity exercise performance.* Res Sports Med, 2008. **16**(3): p. 155-66.
79. Coso, J.D., et al., *Anaerobic performance when rehydrating with water or commercially available sports drinks during prolonged exercise in the heat.* Appl Physiol Nutr Metab, 2008. **33**(2): p. 290-8.
80. Skillen, R.A., et al., *Effects of an amino acid carbohydrate drink on exercise performance after consecutive-day exercise bouts.* Int J Sport Nutr Exerc Metab, 2008. **18**(5): p. 473-92.
81. Green, M.S., et al., *Carbohydrate-protein drinks do not enhance recovery from exercise-induced muscle injury.* Int J Sport Nutr Exerc Metab, 2008. **18**(1): p. 1-18.
82. Valentine, R.J., et al., *Influence of carbohydrate-protein beverage on cycling endurance and indices of muscle disruption.* Int J Sport Nutr Exerc Metab, 2008. **18**(4): p. 363-78.
83. Abbey, E.L. and J.W. Rankin, *Effect of ingesting a honey-sweetened beverage on soccer performance and exercise-induced cytokine response.* Int J Sport Nutr Exerc Metab, 2009. **19**(6): p. 659-72.
84. Anastasiou, C.A., et al., *Sodium replacement and plasma sodium drop during exercise in the heat when fluid intake matches fluid loss.* J Athl Train, 2009. **44**(2): p. 117-23.
85. Osterberg, K.L., C.A. Horswill, and L.B. Baker, *Pregame urine specific gravity and fluid intake by National Basketball Association players during competition.* J Athl Train, 2009. **44**(1): p. 53-7.
86. Schweitzer, G.G., J.D. Smith, and J.D. Lecheminant, *Timing Carbohydrate Beverage Intake During Prolonged Moderate Intensity Exercise Does Not Affect Cycling Performance.* Int J Exerc Sci, 2009. **2**(1): p. 4-18.
87. Valiente, J.S., et al., *Effects of commercially formulated water on the hydration status of dehydrated collegiate wrestlers.* J Strength Cond Res, 2009. **23**(8): p. 2210-6.
88. Currell, K., S. Conway, and A.E. Jeukendrup, *Carbohydrate ingestion improves performance of a new reliable test of soccer performance.* Int J Sport Nutr Exerc Metab, 2009. **19**(1): p. 34-46.
89. Ali, A., et al., *Changes in sensory perception of sports drinks when consumed pre, during and post exercise.* Physiol Behav, 2011. **102**(5): p. 437-43.
90. Armstrong, L.E., et al., *Human hydration indices: acute and longitudinal reference values.* Int J Sport Nutr Exerc Metab, 2010. **20**(2): p. 145-53.
91. Kurdak, S.S., et al., *Hydration and sweating responses to hot-weather football competition.* Scand J Med Sci Sports, 2010. **20 Suppl 3**: p. 133-9.
92. Stanley, J., M. Leveritt, and J.M. Peake, *Thermoregulatory responses to ice-slush beverage ingestion and exercise in the heat.* Eur J Appl Physiol, 2010. **110**(6): p. 1163-73.
93. Millard-Stafford, M.L., M.B. Brown, and T.K. Snow, *Acute carbohydrate ingestion affects lactate response in highly trained swimmers.* Int J Sports Physiol Perform, 2010. **5**(1): p. 42-54.
94. Rutherford, J.A., L.L. Spriet, and T. Stellingwerff, *The effect of acute taurine ingestion on endurance performance and metabolism in well-trained cyclists.* Int J Sport Nutr Exerc Metab, 2010. **20**(4): p. 322-9.
95. Snell, P.G., et al., *Comparative effects of selected non-caffeinated rehydration sports drinks on short-term performance following moderate dehydration.* J Int Soc Sports Nutr, 2010. **7**: p. 28.
96. Stock, M.S., et al., *The effects of adding leucine to pre and postexercise carbohydrate beverages on acute muscle recovery from resistance training.* J Strength Cond Res, 2010. **24**(8): p. 2211-9.

97. Ferguson-Stegall, L., et al., *The effect of a low carbohydrate beverage with added protein on cycling endurance performance in trained athletes.* J Strength Cond Res, 2010. **24**(10): p. 2577-86.
98. Gilson, S.F., et al., *Effects of chocolate milk consumption on markers of muscle recovery following soccer training: a randomized cross-over study.* J Int Soc Sports Nutr, 2010. **7**: p. 19.
99. Alexy, U., et al., *24 h-Sodium excretion and hydration status in children and adolescents--results of the DONALD Study.* Clin Nutr, 2012. **31**(1): p. 78-84.
100. Chia, M. and S. Mukherjee, *Hydration status of heat-acclimatized youth team players during competition.* Science & Sports, 2012. **27**(5): p. e51-e54.
101. Lee, J.K., et al., *Effects of ingesting a sports drink during exercise and recovery on subsequent endurance capacity.* European Journal of Sport Science, 2011. **11**(2): p. 77-86.
102. Silva, M.R., et al., *Effects of a carbohydrate-electrolyte drink on the hydration of young soccer players.* Revista Brasileira de Medicina do Esporte, 2011. **17**: p. 339-343.
103. Spaccarotella, K.J. and W.D. Andzel, *The effects of low fat chocolate milk on postexercise recovery in collegiate athletes.* The Journal of Strength & Conditioning Research, 2011. **25**(12): p. 3456-3460.
104. Blacker, S.D., et al., *The effect of a carbohydrate beverage on the physiological responses during prolonged load carriage.* European journal of applied physiology, 2011. **111**(8): p. 1901-1908.
105. Rowlands, D.S., D.L. Bonetti, and W.G. Hopkins, *Unilateral fluid absorption and effects on peak power after ingestion of commercially available hypotonic, isotonic, and hypertonic sports drinks.* International journal of sport nutrition and exercise metabolism, 2011. **21**(6): p. 480-491.
106. Arnaoutis, G., et al., *Water ingestion improves performance compared with mouth rinse in dehydrated subjects.* Med Sci Sports Exerc, 2012. **44**(1): p. 175-9.
107. Brandenburg, J.P. and M. Gaetz, *Fluid balance of elite female basketball players before and during game play.* International journal of sport nutrition and exercise metabolism, 2012. **22**(5): p. 347-352.
108. Kalman, D.S., et al., *Comparison of coconut water and a carbohydrate-electrolyte sport drink on measures of hydration and physical performance in exercise-trained men.* Journal of the International Society of Sports Nutrition, 2012. **9**(1): p. 1-10.
109. O'Neal, E., S. Poulos, and P. Bishop, *Hydration profile and influence of beverage contents on fluid intake by women during outdoor recreational walking.* European journal of applied physiology, 2012. **112**: p. 3971-3982.
110. Pryor, J.L., S.A. Craig, and T. Swensen, *Effect of betaine supplementation on cycling sprint performance.* J Int Soc Sports Nutr, 2012. **9**(1): p. 12.
111. Watson, P., S.M. Shirreffs, and R.J. Maughan, *Effect of dilute CHO beverages on performance in cool and warm environments.* 2012.
112. Yanagisawa, K., et al., *Electrolyte-carbohydrate beverage prevents water loss in the early stage of high altitude training.* J Med Invest, 2012. **59**(1-2): p. 102-10.
113. Christensen, P.M., M. Nyberg, and J. Bangsbo, *Influence of nitrate supplementation on VO(2) kinetics and endurance of elite cyclists.* Scand J Med Sci Sports, 2013. **23**(1): p. e21-31.
114. Goh, Q., et al., *Recovery from cycling exercise: effects of carbohydrate and protein beverages.* Nutrients, 2012. **4**(7): p. 568-84.
115. Kamijo, Y., et al., *Enhanced renal Na+ reabsorption by carbohydrate in beverages during restitution from thermal and exercise-induced dehydration in men.* Am J Physiol Regul Integr Comp Physiol, 2012. **303**(8): p. R824-33.

116. McRae, K.A. and S.D. Galloway, *Carbohydrate-electrolyte drink ingestion and skill performance during and after 2 hr of indoor tennis match play.* International journal of sport nutrition and exercise metabolism, 2012. **22**(1): p. 38-46.
117. Park, S.G., et al., *Effects of rehydration fluid temperature and composition on body weight retention upon voluntary drinking following exercise-induced dehydration.* Nutrition research and practice, 2012. **6**(2): p. 126-131.
118. Rollo, I., et al., *The effect of carbohydrate-electrolyte beverage drinking strategy on 10-mile running performance.* International journal of sport nutrition and exercise metabolism, 2012. **22**(5): p. 338-346.
119. Rowlands, D.S., et al., *Composite versus single transportable carbohydrate solution enhances race and laboratory cycling performance.* Applied Physiology, Nutrition, and Metabolism, 2012. **37**(3): p. 425-436.
120. Wilkerson, D.P., et al., *Influence of acute dietary nitrate supplementation on 50 mile time trial performance in well-trained cyclists.* European journal of applied physiology, 2012. **112**(12): p. 4127-4134.
121. Price, M.J. and D. Cripps, *The effects of combined glucose-electrolyte and sodium bicarbonate ingestion on prolonged intermittent exercise performance.* Journal of sports sciences, 2012. **30**(10): p. 975-983.
122. Ramos-Jiménez, A., et al., *Acute physiological response to indoor cycling with and without hydration; case and self-control study.* Nutricion hospitalaria, 2013. **28**(5): p. 1487-1493.
123. Baek, S.G., *The effects of different beverage intake on blood components during exercise under high-temperature environment.* J Exerc Rehabil, 2013. **9**(6): p. 511-3.
124. Costa, R.J., et al., *Water and sodium intake habits and status of ultra-endurance runners during a multi-stage ultra-marathon conducted in a hot ambient environment: an observational field based study.* Nutrition journal, 2013. **12**(1): p. 1-16.
125. Moreno, I.L., et al., *Effects of an isotonic beverage on autonomic regulation during and after exercise.* Journal of the International Society of Sports Nutrition, 2013. **10**(1): p. 2.
126. O'Reilly, J. and S.H. Wong, *Effect of a carbohydrate drink on soccer skill performance following a sport-specific training program.* Journal of exercise science & fitness, 2013. **11**(2): p. 95-101.
127. Pross, N., et al., *Influence of progressive fluid restriction on mood and physiological markers of dehydration in women.* British Journal of Nutrition, 2013. **109**(2): p. 313-321.
128. Desbrow, B., et al., *Comparing the rehydration potential of different milk-based drinks to a carbohydrate–electrolyte beverage.* Applied Physiology, Nutrition, and Metabolism, 2014. **39**(12): p. 1366-1372.
129. O'Neal, E.K., et al., *24-h fluid kinetics and perception of sweat losses following a 1-h run in a temperate environment.* Nutrients, 2013. **6**(1): p. 37-49.
130. Roberts, J.D., et al., *Assessing a commercially available sports drink on exogenous carbohydrate oxidation, fluid delivery and sustained exercise performance.* J Int Soc Sports Nutr, 2014. **11**(1): p. 8.
131. Tucker, M.A., et al., *No Change in 24-Hour Hydration Status Following a Moderate Increase in Fluid Consumption.* J Am Coll Nutr, 2016. **35**(4): p. 308-16.
132. Fernandez-Campos, C., A.L. Dengo, and J. Moncada-Jimenez, *Acute Consumption of an Energy Drink Does Not Improve Physical Performance of Female Volleyball Players.* Int J Sport Nutr Exerc Metab, 2015. **25**(3): p. 271-7.
133. Newell, M.L., et al., *The Ingestion of 39 or 64 g.h(-1) of Carbohydrate is Equally Effective at Improving Endurance Exercise Performance in Cyclists.* Int J Sport Nutr Exerc Metab, 2015. **25**(3): p. 285-92.
134. Papacosta, E., G.P. Nassis, and M. Gleeson, *Effects of acute postexercise chocolate milk consumption during intensive judo training on the recovery of salivary hormones,*

salivary SIgA, mood state, muscle soreness, and judo-related performance. Appl Physiol Nutr Metab, 2015. **40**(11): p. 1116-22.

135. Tran Trong, T., et al., *Ingestion of a cold temperature/menthol beverage increases outdoor exercise performance in a hot, humid environment.* PLoS One, 2015. **10**(4): p. e0123815.
136. Malisova, O., et al., *Water Intake and Hydration Indices in Healthy European Adults: The European Hydration Research Study (EHRS).* Nutrients, 2016. **8**(4): p. 204.
137. Schrader, M., et al., *Carbohydrate supplementation stabilises plasma sodium during training with high intensity.* Eur J Appl Physiol, 2016. **116**(9): p. 1841-53.
138. Baguley, B., et al., *The Effect of Ad Libitum Consumption of a Milk-Based Liquid Meal Supplement vs. a Traditional Sports Drink on Fluid Balance After Exercise.* Int J Sport Nutr Exerc Metab, 2016. **26**(4): p. 347-55.
139. Fahey, T.D., et al., *The effects of ingesting polylactate or glucose polymer drinks during prolonged exercise.* Int J Sport Nutr, 1991. **1**(3): p. 249-56.
140. Pryor, J.L., et al., *Hydration Status and Sodium Balance of Endurance Runners Consuming Postexercise Supplements of Varying Nutrient Content.* Int J Sport Nutr Exerc Metab, 2015. **25**(5): p. 471-9.
141. Keen, D.A., E. Constantopoulos, and J.P. Konhilas, *The impact of post-exercise hydration with deep-ocean mineral water on rehydration and exercise performance.* J Int Soc Sports Nutr, 2016. **13**: p. 17.
142. Lambert, G.P., et al., *Effects of carbonated and noncarbonated beverages at specific intervals during treadmill running in the heat.* Int J Sport Nutr, 1993. **3**(2): p. 177-93.
143. Mitchell, J.B., et al., *The effect of volume ingested on rehydration and gastric emptying following exercise-induced dehydration.* Med Sci Sports Exerc, 1994. **26**(9): p. 1135-43.
144. Peart, D.J., A. Hensby, and M.P. Shaw, *Coconut Water Does Not Improve Markers of Hydration During Sub-maximal Exercise and Performance in a Subsequent Time Trial Compared with Water Alone.* Int J Sport Nutr Exerc Metab, 2017. **27**(3): p. 279-284.
145. Peschek, K., et al., *The effects of acute post exercise consumption of two cocoa-based beverages with varying flavanol content on indices of muscle recovery following downhill treadmill running.* Nutrients, 2013. **6**(1): p. 50-62.
146. Upshaw, A.U., et al., *Cycling Time Trial Performance 4 Hours After Glycogen-Lowering Exercise Is Similarly Enhanced by Recovery Nondairy Chocolate Beverages Versus Chocolate Milk.* Int J Sport Nutr Exerc Metab, 2016. **26**(1): p. 65-70.
147. Wilson, P.B. and S.J. Ingraham, *Effects of glucose-fructose versus glucose ingestion on stride characteristics during prolonged treadmill running.* Sports Biomech, 2016. **15**(3): p. 270-82.
148. Briars, G.L., et al., *Swim drink study: a randomised controlled trial of during-exercise rehydration and swimming performance.* BMJ Paediatr Open, 2017. **1**(1): p. e000075.
149. Cebi, M., *The Effect of Sports Drinks and Water Consumption on Electrolyte Levels of Football Players.* Studies on Ethno-Medicine, 2015. **9**(2): p. 197-201.
150. Demirhan, B., et al., *The Effect of Drinking Water and Isotonic Sports Drinks in Elite Wrestlers.* The Anthropologist, 2015. **21**(1-2): p. 213-218.
151. Siow, P.C., W.S.K. Tan, and C.J. Henry, *Impact of Isotonic Beverage on the Hydration Status of Healthy Chinese Adults in Air-Conditioned Environment.* Nutrients, 2017. **9**(3): p. 242.
152. Harper, L.D., et al., *The influence of a 12% carbohydrate-electrolyte beverage on self-paced soccer-specific exercise performance.* J Sci Med Sport, 2017. **20**(12): p. 1123-1129.
153. Palmer, M.S., et al., *Ingesting a sports drink enhances simulated ice hockey performance while reducing perceived effort.* International Journal of Sports Medicine, 2017. **38**(14): p. 1061-1069.
154. Smith, J.W., et al., *Ingestion of an amino acid electrolyte beverage during resistance exercise does not impact fluid shifts into muscle or performance.* Sports, 2017. **5**(2): p. 36.

155. Smith, J.W., et al., *Effects of carbohydrate and branched-chain amino acid beverage ingestion during acute upper body resistance exercise on performance and postexercise hormone response.* Applied Physiology, Nutrition, and Metabolism, 2018. **43**(5): p. 504-509.
156. Rodriguez-Giustiniani, P., et al., *Ingesting a 12% carbohydrate-electrolyte beverage before each half of a soccer match simulation facilitates retention of passing performance and improves high-intensity running capacity in academy players.* International journal of sport nutrition and exercise metabolism, 2019. **29**(4): p. 397-405.
157. Espino-González, E., et al., *The influence of an amaranth-based beverage on cycling performance: a pilot study.* Biotecnia, 2018. **20**(2): p. 31-36.
158. Glace, B.W., I.J. Kremenic, and M.P. McHugh, *Effect of carbohydrate beverage ingestion on central versus peripheral fatigue: a placebo-controlled, randomized trial in cyclists.* Applied Physiology, Nutrition, and Metabolism, 2019. **44**(2): p. 139-147.
159. Ng, J., et al., *Ice slurry ingestion and physiological strain during exercise in non-compensable heat stress.* Aerospace Medicine and Human Performance, 2018. **89**(5): p. 434-441.
160. Schleh, M.W. and C.L. Dumke, *Comparison of sports drink versus oral rehydration solution during exercise in the heat.* Wilderness & Environmental Medicine, 2018. **29**(2): p. 185-193.
161. Onitsuka, S., et al., *Ice slurry ingestion reduces human brain temperature measured using non-invasive magnetic resonance spectroscopy.* Scientific Reports, 2018. **8**(1): p. 2757.
162. Amano, T., et al., *Effects of isomaltulose ingestion on postexercise hydration state and heat loss responses in young men.* Experimental physiology, 2019. **104**(10): p. 1494-1504.
163. Harris, P.R., et al., *Fluid type influences acute hydration and muscle performance recovery in human subjects.* J Int Soc Sports Nutr, 2019. **16**(1): p. 15.
164. Matias, A., et al., *Rehydrating efficacy of maple water after exercise-induced dehydration.* Journal of the International Society of Sports Nutrition, 2019. **16**(1): p. 5.
165. Klimešová, I., et al., *Hydration status and the differences between perceived beverage consumption and objective hydration status indicator in the Czech elite deaf athletes.* Acta Gymnica, 2019. **49**(4): p. 197-202.
166. Berry, C.W., et al., *Hydration efficacy of a milk permeate-based oral hydration solution.* Nutrients, 2020. **12**(5): p. 1502.
167. García-Berger, D., et al., *Effects of skim milk and isotonic drink consumption before exercise on fluid homeostasis and time-trial performance in cyclists: a randomized cross-over study.* Journal of the International Society of Sports Nutrition, 2020. **17**(1): p. 17.
168. McBride, C., et al., *Hydration efficiency of a protein beverage consumed in a bolus vs. metered pattern during recovery.* International Journal of Exercise Science, 2020. **13**(2): p. 1476.
169. Bradbury, K.E., et al., *Effects of carbohydrate supplementation on aerobic exercise performance during acute high altitude exposure and after 22 days of acclimatization and energy deficit.* Journal of the International Society of Sports Nutrition, 2020. **17**(1): p. 4.
170. Fan, P.W., S.F. Burns, and J.K.W. Lee, *Efficacy of ingesting an oral rehydration solution after exercise on fluid balance and endurance performance.* Nutrients, 2020. **12**(12): p. 3826.
171. Flood, T.R., et al., *Addition of pectin-alginate to a carbohydrate beverage does not maintain gastrointestinal barrier function during exercise in hot-humid conditions better than carbohydrate ingestion alone.* Applied Physiology, Nutrition, and Metabolism, 2020. **45**(10): p. 1145-1155.

172. Kitson, O., et al., *Sensory Perception of an Oral Rehydration Solution during Exercise in the Heat.* Nutrients, 2021. **13**(10): p. 3313.
173. Rollo, I., et al., *Fluid balance, sweat Na+ losses, and carbohydrate intake of elite male soccer players in response to low and high training intensities in cool and hot environments.* Nutrients, 2021. **13**(2): p. 401.
174. Nakamura, M., et al., *Effect of ice slurry ingestion on core temperature and blood pressure response after exercise in a hot environment.* Journal of Thermal Biology, 2021. **98**: p. 102922.
175. Otsuka, J., et al., *Effects of isomaltulose ingestion on thermoregulatory responses during exercise in a hot environment.* International Journal of Environmental Research and Public Health, 2021. **18**(11): p. 5760.
176. Amano, T., et al., *Comparison of hydration efficacy of carbohydrate-electrolytes beverages consisting of isomaltulose and sucrose in healthy young adults: A randomized crossover trial.* Physiology & Behavior, 2022. **249**: p. 113770.
177. Bechke, E.E., et al., *Utility of an isotonic beverage on hydration status and cardiovascular alterations.* Nutrients, 2022. **14**(6): p. 1286.
178. Heileson, J.L., et al., *Comparison of a Sucrose-Based and Rice-Based Sports Beverage on Hydration Status During a 19.3-km Foot March in ROTC Cadets.* Journal of Strength and Conditioning Research, 2022. **36**(4): p. 1105-1110.
179. Yun, H.J., et al., *The effects of fluid absorption and plasma volume changes in athletes following consumption of various beverages.* BMC Sports Sci Med Rehabil, 2022. **14**(1): p. 207.
180. Capitán-Jiménez, C. and L.F. Aragón-Vargas, *Post-exercise voluntary drinking cessation is associated with the normalization of plasma osmolality and thirst perception, but not of urine indicators or net fluid balance.* Nutrients, 2022. **14**(19): p. 4188.
181. Molaeikhaletabadi, M., et al., *Short-Term effects of Low-Fat chocolate milk on delayed onset muscle soreness and performance in players on a women's university badminton team.* International journal of environmental research and public health, 2022. **19**(6): p. 3677.
182. Morito, A., et al., *Ice slurry ingestion improves physical performance during high-intensity intermittent exercise in a hot environment.* PloS one, 2022. **17**(9): p. e0274584.
183. Naito, T., et al., *Pre-cooling with ingesting a high-carbohydrate ice slurry on thermoregulatory responses and subcutaneous interstitial fluid glucose during heat exposure.* Journal of Physiological Anthropology, 2022. **41**(1): p. 34.
184. Takada, S., et al., *Effects of ingestion of isomaltulose beverage on plasma volume and thermoregulatory responses during exercise in the heat.* European Journal of Applied Physiology, 2022. **122**(12): p. 2615-2626.
185. Davies, A., et al., *Limited Effect of Dehydrating via Active vs. Passive Heat Stress on Plasma Volume or Osmolality, Relative to the Effect of These Stressors per Se.* Nutrients, 2023. **15**(4): p. 904.
186. Goldstein, E.R., et al., *Carbohydrate-Protein drink is effective for restoring endurance capacity in masters class athletes after a two-Hour recovery.* Journal of the International Society of Sports Nutrition, 2023. **20**(1): p. 2178858.
187. Ng, J. and J.E. Wingo, *Effect of Ice Slurry Beverages on Voluntary Fluid Intake and Exercise Performance.* Journal of Strength and Conditioning Research, 2023. **37**(6): p. e376-e383.
188. Bachle, L., et al., *The effect of fluid replacement on endurance performance.* The Journal of Strength & Conditioning Research, 2001. **15**(2): p. 217-224.
