# Supplementary material for: Trends and Missing Links in (De)Hydration Research: A Narrative Review
Source: Nutrients. 2024 May 30;16(11):1709. doi: 10.3390/nu16111709 (PMC11174495; doi:10.3390/nu16111709)
Supplement: Supplementary file 1 [file nutrients-16-01709-s001.zip › OSF2 Table S2 - Publication and Participant-level details.pdf]

Supplementary Table S2. Publication and participant-level details

Study (year)	Country(s)	Study design Interventional (randomized or non-randomized) / observational	Sample size (*n*)	Sex (*n*)	Mean age (age range)	Athlete/non-athlete tier level
Abbey *et.al.* (2009)	USA	Interventional: Randomized	10	10 ♂	22.5 (18-30)	Athlete Tier 2
Alexy *et al.* (2011)	Germany	Observational	499	249 ♂ 250 ♀	NR (4-18)	Non-Athlete NR
Ali *et al.* (2010)	New Zealand	Interventional: Non-Randomized	14	14 ♂	24.4	Non-Athlete Tier 1
Amano *et al.* (2019)	Japan	Interventional: Randomized	10	10 ♂	21.2	Non-Athlete Tier 1
Amano *et al.* (2022)	Japan	Interventional: randomised	13	7 ♀ 6 ♂	21.8	Non-Athlete NR
Anastasiou *et al.* (2009)	Greece	Interventional: Randomized	13	13 ♂	24.5	Non-Athlete Tier 1
Armstrong *et al.* (2010)	Greece	Observational	59	59 ♂	22	Non-Athlete Tier 1
Arnaoutis *et al.* (2012)[1]	Greece	Interventional: Randomized	10	10 ♂	25.9	Athlete Tier 2
Bachle *et al.* (1998)	USA	Interventional: Randomized	10	6 ♀ 4 ♂	29	Non-Athlete Tier 1
Bechke *et al.* (2022)	USA	Interventional: Randomized	22	11 ♀ 11 ♂	27	Non-Athlete NR
Berry *et al.* (2020)	USA	Interventional: Randomized	12	6 ♀ 6 ♂	23 (20-26)	Non-Athlete NR

[1] This trial is not colored because it did not mention a hydration protocol (only described a dehydration protocol).

Brandenburg *et al.* (2012)	Canada	Observational	17	17 ♀	24.2	Athlete<br>Tier 3
Briars *et al.* (2017)	UK	Interventional: Randomized	19	10 ♂<br>9 ♀	13<br>(11-17)	Athlete<br>Tier 2
Mehmet Cebi (2017)	Turkey	Interventional: Non-Randomized	20	NR	20.5	Athlete<br>Tier 2
Chia *et al.* (2011)	Singapore	Observational	40	40 ♂	12.3	Non-Athlete<br>Tier 1
Clarke *et al.* (2019)	USA	Interventional: Randomized	24	12 ♀<br>12 ♂	NR<br>(18-30<br>60-85)	Non-Athlete<br>NR
Davies *et al.* (2023)	New Zealand	Interventional: Non-Randomized	12	5 ♀<br>7 ♂	33.5	Non-Athlete<br>Tier 1
Vieira de Carvalho *et al.* (2007)	Brazil	Interventional: Randomized	26	26 ♂	18.88	Non-Athlete<br>NR
Demirhan *et al.* (2017)	Turkey	Interventional: Non-Randomized	18	NR	NR<br>(18-20)	Athlete<br>Tier 4
Desbrow *et al.* (2014)	Australia	Interventional: Non-Randomized	15	15 ♂	24.9	Non-Athlete<br>NR
García-Berger *et al.* (2020)	Chile	Interventional: Randomized	9	9 ♂	26.8	Athlete<br>Tier 3
D.B. Goulet *et al.* (2008)	Canada	Interventional: Randomized	6	5 ♂<br>1 ♀	36.5	Athlete<br>NR
R. Harris *et al.* (2019)	USA	Interventional: Non-Randomized	17	9 ♂<br>8 ♀	22.9	Non-Athlete<br>Tier 1
Heileson *et al.* (2022)	USA	Interventional: Randomized	11	11 ♂<br>7 ♀	20.2	Non-Athlete<br>NR
J. Hill et. Al (2008)	Australia	Interventional: Randomized	34	18 ♂<br>16 ♀	26.25	Non-Athlete<br>NR

I Ismail *et al.* (2007)	Malaysia	Interventional: Randomized	10	10 ♂	20.7	Non-Athlete<br>Tier 1
S Kalman *et al.* (2012)	USA	Interventional: Randomized	12	12 ♂	26.6<br>(21-35)	Non-Athlete<br>Tier 1
Malisova *et al.* (2016)	Spain,<br>Germany, or<br>Greece	Observational	573	293 ♂<br>280 ♀	39<br>(20-60)	Non-Athlete<br>NR
Kitson *et al.* (2021)	New Zealand	Interventional: Randomized	27	13 ♂<br>14 ♀	25	Non-Athlete<br>Tier 1
Kurdak *et al.* (2010)	Turkey	Interventional: Non-Randomized	22	NR	20	Athlete<br>NR
K. W. Lee *et al.* (2011)	Singapore	Interventional: Randomized	12	12 ♂	24.3	Non-Athlete<br>Tier 1
Matias *et al.* (2019)	USA	Interventional: Non-Randomized	26	13 ♂<br>13 ♀	22	Non-Athlete<br>Tier 1
J. Maughan *et al.*<br>(2007)	UK	Interventional: Non-Randomized	20	NR	21	Athlete<br>Tier 3
McBride *et al.* (2020)	USA	Interventional: Non-Randomized	10	10 ♂	23.7	Non-Athlete<br>Tier 1
Meyer *et al.* (1994)	Canada	Interventional: Randomized	24	10 ♂<br>14 ♀	NR	Non-Athlete<br>Tier 1
Millard-Stafford *et al.*<br>(1995)	USA	Interventional: Non-Randomized	12	6 ♂<br>6 ♀	30	Athlete<br>Tier 3
L. Millard-Stafford *et al.*<br>(2005)	USA	Interventional: Non-Randomized	10	10 ♂	23.7	Athlete<br>Tier 3
Mitchell *et al.* (2000)	USA	Interventional: Randomized	10	10 ♂	27.5	Athlete<br>Tier 2
Mora-Rodriguez *et al.*<br>(2016)	Germany<br>Spain<br>Greece	Observational	573	NR ♂<br>NR ♀	NR<br>(20-60)	Non-Athlete<br>NR

K. O'Neal *et al.* (2014)	USA	Interventional: Non-Randomized	20	12 ♂<br>8 ♀	20	Non-Athlete<br>Tier 1<br>Athlete<br>Tier 3
K. O'Neal *et al.* (2012)	USA	Interventional: Non-Randomized	27	27 ♀	23.9	Non-Athlete<br>Tier 1
L. Osterberg *et al.* (2009)	USA	Interventional: Non-Randomized	29	NR	NR	Athlete<br>Tier 3
Luke Pryor *et al.* (2012)	USA	Interventional: Randomized	16	9 ♂<br>7 ♀	19	Non-Athlete<br>Tier 1
Ramos-Jiménez *et al.* (2013)	Mexico	Interventional: Non-Randomized	14	14 ♂	32	Non-Athlete<br>Tier 1
M. Rivera-Brown *et al.* (1999)	USA	Interventional: Randomized	12	12 ♂	13.4<br>(11-14)	Athlete<br>Tier 2
M. Rivera-Brown *et al.* (2008)	USA	Interventional: Randomized	12	12 ♀	10.6<br>(9-12)	Athlete<br>Tier 2
D Roberts *et al.* (2014)	UK	Interventional: Randomized	14	14 ♂	31.79	Non-Athlete<br>Tier 1
Rodriguez-Giustiniani *et al.* (2018 accepted)	UK	Interventional: Randomized	18	18 ♂	18	Athlete<br>Tier 4
Rollo *et al.* (2021)	UK<br>Spain	Observational	14	14 ♂	24	Athlete<br>Tier 4
J. Saunders *et al.* (2004)	USA	Interventional: Randomized	15	15 ♂	20.9	Athlete<br>Tier 2
Schrader *et al.* (2016)	Germany	Interventional: Non-Randomized	10	10 ♂	26.18	Non-Athlete<br>Tier 1<br>Athlete<br>Tier 2
G. Schweitzer *et. al* (NR)	USA	Interventional: Non-Randomized	10	8 ♂<br>2 ♀	30.1	Athlete<br>Tier 2

M. Shirreffs *et. al* (NR)	UK	Interventional: Randomized	11	5 ♂<br>6 ♀	24	Non-Athlete<br>Tier 1
Reis Silva *et. al* (2011)	Brazil	Interventional: Non-Randomized	9	9 ♂	NR	Athlete<br>Tier 2
Ching Siow et al (2017)	Singapore	Interventional: Randomized	49	49 ♂	25	Non-Athlete<br>NR
J. Spaccarotella *et. al* (2011)	USA	Interventional: Randomized	13	5 ♂<br>8 ♀	19.5	Athlete<br>Tier 3
Stanley *et. al* (2010)	Australia	Interventional: Randomized	10	10 ♂	30	Athlete<br>Tier 2
MF Sun *et. al* (2008)	Singapore	Interventional: Non-Randomized	10	5 ♂<br>5 ♀	21.9	Athlete<br>Tier 4
A. Tucker *et. al* (2015)	USA	Interventional: Randomized	35	35 ♂	23.75	Non-Athlete<br>Tier 1
Valiente *et. al* (2009)	USA	Interventional: Randomized	21	21 ♂	19.4	Athlete<br>Tier 4
Vrijens *et. al* (1999)	New Zealand	Interventional: Randomized	10	10 ♂	24.8	Athlete<br>Tier 2
Watson P *et. al* (2012)	UK	Interventional: Randomized	24	24 ♂	21.5	Non-Athlete<br>Tier 1
Wilkan *et al.* (1996)	UK	Interventional: Randomized	12	12 ♂	10.42	Non-Athlete<br>Tier 1
Wilk *et. al* (1998)	Canada	Interventional: Randomized	12	12 ♂	11.17	Non-Athlete<br>Tier 1<br>athelete<br>Tier 2
Wilk *et. al* (2007)	Canada	Interventional: Randomized	12	12 ♀	10.6	Non-Athlete<br>Tier 1

E. Wing *et al.* (2004)	USA	Interventional: Randomized	12	12 ♂	24.5	Athlete<br>Tier 2
Yanagisawa *et al.* (2012)	Japan	Interventional: Non-Randomized	16	16 ♂	21.3	Athlete<br>Tier 2
Yun *et al.* (2022)	Korea	Interventional: Randomized	31	NR	20.1	Athlete<br>NR
Backhouse *et al.* (2005)	UK	Interventional: Randomized	9	9 ♂	25	Athlete<br>Tier 2
Soon Gi Baek *et. al* (NR)	Korea	Interventional: Non-Randomized	10	NR	22.5	Non-Athlete<br>NR
J. Baguley *et. al* (2016)	Australia	Interventional: Randomized	7	7 ♂	22.3	Non-Athlete<br>Tier 1
P. Bailey *et al.* (2008)	USA	Interventional: Randomized	10	10 ♂	23	Non-Athlete<br>Tier 1
Barr SI *et al.* (1991)	USA	Interventional: Randomized	8	5 ♂<br>3 ♀	28	Athlete<br>Tier 3
J. BATY *et al.* (2007)	USA	Interventional: Non-Randomized	34	34 ♂	21.5	Non-Athlete<br>NR
D. Blacker *et al.* (2011)	UK	Interventional: Randomized	10	10 ♂	28	Non-Athlete<br>NR
E. Bradbury *et al.* (2020)	USA	Interventional: Randomized	14	14 ♂	NR<br>(18-42)	Non-Athlete<br>Tier 1
Capitán-Jiménez *et al.* (2022)	Costa Rica	Interventional: Randomized	9	9 ♂	26.6	Non-Athlete<br>Tier 1
Carter JE *et al.* (1989)	USA	Interventional: Randomized	7	7 ♂	NR<br>(20-33)	Non-Athlete<br>NR
M. Christensen *et al.* (2012)	Denmark	Interventional: Non-Randomized	10	10 ♂	29	Athlete<br>Tier 4

J. Clapp *et. al* (2000)	USA	Interventional: Randomized	18	18 ♂	24.6<br>(19-32)	Non-Athlete<br>NR
JS Costa *et al.* (2013)	UK	Observational	86	51 ♂<br>35 ♀	38	Non-Athlete<br>Tier 1
Criswell *et al.* (1992)	USA	Interventional: Non-Randomized	6	6 ♂	23	Athlete<br>NR
Currell *et al.* (2009)	UK	Interventional: Randomized	11	11 ♂	21.4	Non-Athlete<br>at least Tier 1
Currell *et al.* (2008)	UK	Interventional: Randomized	8	8 ♂	32	Athlete<br>NR
Davis *et al.* (1990)	USA	Interventional: Non-Randomized	8	8 ♂	NR	Non-Athlete<br>NR
M. Davis *et al.* (1988)	USA	Interventional: Non-Randomized	15	15 ♂	NR<br>(20-31)	Athlete<br>Tier 2
Mark Davis *et al.* (1997)	USA	Interventional: Non-Randomized	16	9 ♂<br>7 ♀	24.05	Non-Athlete<br>Tier 1
Mark Davis *et al.* (1998)	USA	Interventional: Non-Randomized	19	19 ♂	NR<br>(16-26)	Athlete<br>Tier 3
M. Davis *et al.* (1999)	USA	Interventional: Non-Randomized	8	3 ♂<br>5 ♀	NR	Non-Athlete<br>Tier 1
Davison *et al.* (2008)	UK	Interventional: Randomized	10	10 ♂	20	Non-Athlete<br>Tier 1
Del coso *et al.* (2008)	Spain	Interventional: Randomized	7	7 ♂	NR	Athlete<br>NR
Espino-González *et al.* (2018)	Mexico	Interventional: Randomized	6	5 ♂<br>1 ♀	NR<br>(20-32)	Athlete<br>Tier 3
D. Fahey *et al.* (1991)	USA	Interventional: Non-Randomized	5	5 ♂	25.6	Athlete<br>NR

Weiping Fan *et al.* (2020)	Singapore	Interventional: Randomized	9	9 ♂	24	Non-Athlete<br>Tier 1
Mark A *et al.* (1996)	Australia	Interventional: Randomized	18	10 ♂<br>2 ♀	27.3	Athlete<br>Tier 2
Fernández-Campos *et al.* (2015)	USA	Interventional: Randomized	20	20 ♀	22.3	Athlete<br>Tier 4
W. Glace *et al.* (2018)	USA	Interventional: Non-Randomized	20	10 ♂<br>10 ♀	35	Athlete<br>Tier 2
Glickman-Weiss *et al.* (1995)	USA	Interventional: Non-Randomized	7	7 ♂	34.7	Non-Athlete<br>NR
Goh *et al.* (2012)	USA	Interventional: Randomized	12	12 ♂	25	Athlete<br>Tier 2
R. Goldstein *et al.* (2023)	USA	Interventional: Randomized	22	22 ♂	49.1	Athlete<br>NR
Harper *et al.* (2015)	UK	Interventional: Randomized	8	8 ♂	16	Athlete<br>Tier 4
Harper *et al.* (2017)	UK	Interventional: Randomized	15	NR	22	Athlete<br>Tier 3
S. Hickey *et. al* (NR)	USA	Interventional: Randomized	8	8 ♂	34	Athlete<br>Tier 3
HoRIE *et al.* (2003)	Japan	Interventional: Non-Randomized	8	8 ♂	46.3<br>(41-56)	Non-Athlete<br>NR
Pryor *et al.* (NR)	USA	Interventional: Randomized	8	8 ♂	22	Athlete<br>Tier 3
Kamijo *et al.* (2012)	Japan	Interventional: Randomized	7	7 ♂	25	Non-Athlete<br>Tier 1
Keen *et al.* (2016)	USA	Interventional: Randomized	20	NR	23	Athlete<br>NR

Klimešová *et al.* (2019)	Czech Republic	Observational	62	62 ♂	25.8	Athlete<br>Tier 2
Koulmann *et al.* (1997)	France	Interventional: Randomized	6	6 ♂	27	Non-Athlete<br>Tier 1
Lambert *et al.* (1992)	USA	Interventional: Randomized	8	8 ♂	28	Non-Athlete<br>NR
Lambert *et al.* (NR)	USA	Interventional: Non-Randomized	8	8 ♂	29.5	Athlete<br>Tier 2
Lyons *et al.* (1990)	USA	Interventional: Randomized	6	4 ♂<br>2 ♀	26.2	Non-Athlete<br>NR
Maughan *et al.* (1994)	Scotland	Interventional: Randomized	8	8 ♂	22	Non-Athlete<br>Tier 1
A. McRae *et al.* (NR)	Scotland	Interventional: Randomized	22	15 ♂<br>7 ♀	22	Athlete<br>Tier 3
Millard-Stafford *et al.* (NR)	USA	Interventional: Randomized	12	12 ♂	29.8	Athlete<br>Tier 3
Millard-Stafford *et al.* (2010)	USA	Interventional: Non-Randomized	23	12 ♂<br>11 ♀	20.05	Athlete<br>Tier 2-5
Millard-Stafford *et al.* (1992)	USA	Interventional: Non-Randomized	8	8 ♂	32.1	Athlete<br>Tier 3
Mitchell *et al.* (2000)	USA	Interventional: Randomized	10	10 ♂	29.5	Athlete<br>Tier 2
Mitchell JB *et al.* (2016)	USA	Interventional: Randomized	9	9 ♂	27.3	Non-Athlete<br>Tier 1
Molaeikhaletabadi *et al.* (2022)	Iran	Interventional: Randomized	7	7 ♀	23	Athlete<br>Tier 2
Moreno *et al.* (2013)	Brazil	Interventional: Non-Randomized	31	31 ♂	21.5<br>(18-25)	Non-Athlete<br>Tier 1

Morito *et al.* (2022)	Japan	Interventional: Randomized	12	12 ♂	19.8<br>(18-22)	Athlete<br>Tier 2
Murray *et al.* (1989)	USA	Interventional: Non-Randomized	12	7 ♂<br>5 ♀	30.7	Non-Athlete<br>Tier 1
Naito *et al.* (2022)	Japan	Interventional: Non-Randomized	7	7 ♂	30.4	Non-Athlete<br>Tier 1
Nakamura *et al.* (2021)	Japan	Interventional: Randomized	7	7 ♂	21	Athlete<br>NR
Newell *et al.* (2015)	UK	Interventional: Randomized	20	20 ♂	34	Athlete<br>Tier 2
Jason *et al.* (2023)	USA	Interventional: Randomized	8	7 ♂<br>1 ♀	24	Non-Athlete<br>Tier 1
Jason *et al.* (2018)	USA	Interventional: Randomized	8	8 ♂	21	Non-Athlete<br>Tier 1
Niles *et al.* (2001)	USA	Interventional: Non-Randomized	10	10 ♂	27.4<br>(24-34)	Athlete<br>NR
O'Reilly *et al.* (2013)	China	Interventional: Randomized	7	7 ♂	23	Athlete<br>Tier 3
Otskua *et al.* (2021)	Japan	Interventional: Randomized	10	10 ♂	20.4	Athlete<br>Tier 2
Owen MD *et al.* (1986)	USA	Interventional: Non-Randomized	5	5 ♂	33.6	Athlete<br>NR
Palmer *et al.* (2017)	Canada	Interventional: Randomized	7	7 ♂	21.3	Non-Athlete<br>NR
Papacosta *et al.* (2015)	Cyprus	Interventional: Non-Randomized	12	12 ♂	19	Athlete<br>Tier 3
Park *et al.* (2012)	Korea	Interventional: Randomized	8	8 ♂	21.5	Non-Athlete<br>NR

Peart *et al.* (2016)	UK	Interventional: Randomized	10	10 ♂	27.9	Non-Athlete<br>Tier 3
Peschek *et al.* (2014)	USA	Interventional: Randomized	8	8 ♂	NR<br>(18-44)	Athlete<br>Tier 2
Powers *et al.* (1990)	USA	Interventional: Randomized	9	NR	25.9	Athlete<br>Tier 3
Pross *et al.* (2013)	France	Interventional: Randomized	20	20 ♀	25	Non-Athlete<br>NR
Rollo *et al.* (2012)	UK	Interventional: Randomized	9	9 ♂	31	Athlete<br>Tier 2
Rowlands (2011)	New Zealand	Interventional: Randomized	11	11 ♂	29	Athlete<br>Tier 3
Rowlands (2012)	New Zealand	Interventional: Randomized	26	22 ♂<br>3 ♀	33	Athlete<br>Tier 3
Rutherford *et al.* (2010)	Canada	Interventional: Randomized	11	11 ♂	27.2	Athlete<br>Tier 2
Ryan *et al.* (1991)	USA	Interventional: Non-Randomized	9	9 ♂	NR<br>(19-40)	Non-Athlete<br>NR
Aoki *et al.* (2003)	Brazil	Interventional: Randomized	6	6 ♀	22.4	Non-Athlete<br>Tier 1
Sanders *et al.* (1999)	South Africa	Interventional: Randomized	6	6 ♂	24	Athlete<br>Tier 3
Schleh *et al.* (2018)	USA	Interventional: Randomized	10	10 ♂	22.5	Non-Athlete<br>Tier 1
DS *et al.* (1991)	Israel	Interventional: Randomized	43	43 ♂	20.1	Non-Athlete<br>Tier 1
Shirreffs *et al.* (2007)	Costa Rica	Interventional: Randomized	8	4 ♂<br>4 ♀	23	Non-Athlete<br>Tier 1

Skillen *et al.* (2008)	USA	Interventional: Randomized	12	12 ♂	28.5	Athlete<br>Tier 3
Smith *et al.* (2017)	USA	Interventional: Randomized	18	18 ♂	23.36	Non-Athlete<br>Tier 1
Smith *et al.* (2017)	USA	Interventional: Randomized	13	13 ♂	23	Non-Athlete<br>Tier 1
Snell wt al. (2010)	USA	Interventional: Non-Randomized	8	8 ♂	28.5	Athlete<br>NR
Matt S. *et al.* (2010)	USA	Interventional: Randomized	20	17 ♂<br>3 ♀	22.25	Athlete<br>NR
Takada *et al.* (2022)	Japan	Interventional: Randomized	12	12 ♂	21.1	Athlete<br>Tier 2
Trong *et al.* (2015)	Guadalupe	Interventional: Randomized	10	10 ♂	41	Athlete<br>Tier 3
Upshaw *et al.* (2016)	Canada	Interventional: Non-Randomized	8	8 ♂	21.8	Athlete<br>Tier 2
Utter AC *et al.* (2002)	USA	Interventional: Randomized	98	NR ♂<br>NR ♀	41.95	Athlete<br>NR
Wilkerson *et al.* (2012)	UK	Interventional: Randomized	8	8 ♂	31	Athlete<br>Tier 2
Wilson *et al.* (2016)	USA	Interventional: Randomized	20	14 ♂<br>6 ♀	33.55	Athlete<br>Tier 2
Wong *et al.* (1997)	Japan	Interventional: Randomized	7	2 ♂<br>5 ♀	19.8	Athlete<br>Tier 3
El-Sayed *et al.* (1996)	UK	Interventional: Randomized	8	8 ♂	24	Athlete<br>Tier 3
Febbraio *et al.* (2000)	Australia	Interventional: Randomized	7	7 ♂	26.9	Athlete<br>Tier 2

Ferguson-Stegall *et al.* (2010)	USA	Interventional: Randomized	15	8 ♂<br>7 ♀	28.7	Athlete<br>Tier 2
Finn *et al.* (2004)	USA	Interventional: Randomized	15	NR	20.8	Athlete<br>Tier 4
Flood *et al.* (2020)	UK	Interventional: Randomized	14	7 ♂<br>7 ♀	25	Non-Athlete<br>Tier 1
F Gilson *et al.* (2010)	USA	Interventional: Randomized	13	13 ♂	NR	Athlete<br>Tier 4
Green *et al.* (2008)	USA	Interventional: Non-Randomized	16	6 ♂<br>6 ♀	24.6	Non-Athlete<br>Tier 1
Luden *et al.* (2007)	USA	Interventional: Randomized	23	11 ♂<br>12 ♀	19.35	Athlete<br>Tier 4
Onitsuka *et al.* (2018)	Japan	Interventional: Non-Randomized	8	8 ♂	26.9	Non-Athlete<br>NR
Palmer *et al.* (1998)	South Africa	Interventional: Randomized	14	11 ♂<br>3 ♀	23.2	Athlete<br>NR
Price *et al.* (2012)	UK	Interventional: Non-Randomized	9	9 ♂	25.4	Non-Athlete<br>Tier 1
Alan C *et al.* (2005)	USA	Interventional: Randomized	30	30 ♂	21.45	Athlete<br>NR
Utter AC *et al.* (2004)	USA	Interventional: Randomized	16	NR	50	Athlete<br>Tier 2
C. utter *et al.* (1999)	USA	Interventional: Randomized	10	8 ♂<br>2 ♀	34	Athlete<br>Tier 3
J. Valentine *et al.* (2008)	USA	Interventional: Non-Randomized	11	11 ♂	20.8	Athlete<br>Tier 2
Warber *et al.* (2000)	USA	Interventional: Non-Randomized	14	14 ♂	26	Non-Athlete<br>NR

Widrick *et al.* (1993)	USA	Interventional: Randomized	8	8 ♂	26	Athlete<br>Tier 2
Williams *et al.* (2003)	USA	Interventional: Randomized	8	8 ♂	24.3	Athlete<br>Tier 2
Wojcik *et al.* (2001)	USA	Interventional: Randomized	26	26 ♂	23.5	Non-Athlete<br>NR

Legend: **Green** stands for studies studying the effects of different beverages, **orange** for similar beverages but different composition, **blue** for similar beverages at different temperatures, **yellow** for beverages taken at different kinetics, **gray** for others, and **pink** for observational studies. **Red** represents one trial where two of the mentioned strategies were applied: dehydration protocol and beverages taken at different kinetics. ♂ - Male, ♀ - Female, NA – non-applicable, NR – non-reported.

Reporting details: Sample size refers only to subjects assessed on trial. If no common value for sample size was provided by authors, a counting of all participants was made, despite different study conditions. Age is reported in years and as the mean age of all participants (when authors reported age separately for different groups, the mean of those values was calculated and presented herein). Subjects meeting requirements for at least Tier 2 were considered athletes.
