# Supplementary material for: Trends and Missing Links in (De)Hydration Research: A Narrative Review
Source: Nutrients. 2024 May 30;16(11):1709. doi: 10.3390/nu16111709 (PMC11174495; doi:10.3390/nu16111709)
Supplement: Supplementary file 1 [file nutrients-16-01709-s001.zip › OSF3 Table S3 - Intervention-level details.pdf]

Supplementary Table S3. Intervention-level details

**Study (year)**	**Sport(s)**	**Exercise length** (min/set/reps/km/ex) **Dehydration** - % change in body weight	**Intake strategy** (mL/bd loss/% bd loss/% sweat loss/free/mL per bd/% bd)	Environmental conditons ***Temperature***	Environmental conditons ***Relative humidity***	**Within-season**
Abbey *et.al.* (2009)	Soccer	75 + ex	-	NR	NR	Pre-season
Alexy *et al.* (2011)	NA	-	-	NR	NR	NA
Ali *et al.* (2010)	Treadmill Running	60	300	RT	Normal	NA
Amano *et al.* (2019)	Cycling	105<br>2%	bd loss	RT	Normal	NA
Amano *et al.* (2022)	NA	-	1000	RT	Normal	NA
Anastasiou *et al.* (2009)	NA	225<br>8 sets 30 reps	bd loss	RT	NR	NA
Armstrong *et al.* (2010)	NA	-	-	NA	NA	NA
Arnaoutis *et al.* (2012)	Cycling	NR<br>2%	-	Hot	NR	NR
Bachle *et al.* (1998)	Cycling	60	1200	NR	NR	NA
Bechke *et al.* (2022)	NA	-	1000	NA	NA	NA
Berry *et al.* (2020)	NA	-	1000	RT	Below	NA

Brandenburg *et al.* (2012)	Basketball	40	free	RT	Normal	Competitive phase
Briars *et al.* (2017)	Swimming	105	1000	NR	NR	NR
Mehmet Cebi (2017)	Football	90	1000	RT	NR	NR
Chia *et al.* (2011)	Hockey	6	free	Hot	Above	NA
Clarke *et al.* (2019)	NA	-	1500	NA	NA	NA
Davies *et al.* (2023)	Cycling	4<br>3%	free	RT	Normal	NA
Vieira de Carvalho *et al.* (2007)	Walking	16 km	1000	RT	Above	NA
Demirhan *et al.* (2017)	Wrestling	45	800	NR	NR	NR
Desbrow *et al.* (2014)	Cycling	1.8%	150% bd loss	RT	Above	NA
García-Berger *et al.* (2020)	Cycling	18.6 km	1200	RT	Normal	NR
D.B. Goulet *et al.* (2008)	Cycling	90 + ex	85349	RT	Normal	NR
R. Harris *et al.* (2019)	Cycling	3%	bd loss	RT	NR	NA
Heileson *et al.* (2022)	Walking	19.3 km	2840	Cool	Normal	NA
J. Hill et. Al (2008)	Walking	60	300	NR	NR	NA

I Ismail *et al.* (2007)	Running	90<br>3%	120% bd loss	Hot<br>RT	Normal<br>Above	NA
S Kalman *et al.* (2012)	Treadmill	90<br>26 + ex<br>2 to 3%	125% bd loss	Hot<br>RT	Normal	NA
Malisova *et al.* (2016)	NA	-	-	NR	NR	NA
Kitson *et al.* (2021)	Cycling	60	-	Hot	Normal	NA
Kurdak *et al.* (2010)	Football	90	free	Hot	Above	NA
K. W. Lee *et al.* (2011)	Cycling	75<br>ex	150% sweat loss	Hot	Above	NA
Matias *et al.* (2019)	Cycling	–<br>2%	1000	RT	Normal	NA
J. Maughan *et al.* (2007)	Football	90	1000	Cool	Normal	Competitive phase
MCBRIDE *et al.* (2020)	Running	120<br>2 to 2,5%	125% bd loss	NR	NR	NA
Meyer *et al.* (1994)	Cycling	60<br>2 to 2,5%	free	Hot	Below	NA
Millard-Stafford *et al.* (1995)	Running	40 km	2400 + free	RT	Above	NR
L. Millard-Stafford *et al.* (2005)	Running	32 km	1900	RT	NR	NR
MITCHELL *et al.* (2000)	Cycling	140 min<br>2,5%	7370	Hot<br>RT	Normal	NR
Mora-Rodriguez *et al.* (2016)	NA	-	-	NA	NA	NA

K. O'Neal *et al.* (2014)	Running	60	705 + free	RT	Normal	NR
K. O'Neal *et al.* (2012)	Walking	60	free	RT	NR	NA
L. Osterberg *et al.* (2009)	Basketball	40	free	RT	Below	Competitive phase
Luke Pryor *et al.* (2012)	Cycling	16	4137	NR	NR	NA
Ramos-Jiménez *et al.* (2013)	Cycling	90	1679	RT	Below	NA
M. RIVERA-BROWN *et al.* (1999)	Cycling	80	free	Hot	Normal	NR
M. Rivera-Brown *et al.* (2008)	Cycling	80	free	Hot	Above	NR
D Roberts *et al.* (2014)	Cycling	150<br>60 km	-	RT	Below<br>Normal	NA
Rodriguez-Giustiniani *et al.* (2018 accepted)	Soccer	90<br>ex	500	Cool	Normal	NR
Rollo *et al.* (2021)	Soccer	65	free	Cool<br>RT	Above<br>Normal	Competitive phase
J. SAUNDERS *et al.* (2004)	Cycling	ex	-	NR	NR	NR
Schrader *et al.* (2016)	Cycling	ex	-	RT	NR	NA
G. SCHWEITZER *et. al* (NR)	Cycling	140	-	NR	NR	NR
M. Shirreffs *et. al* (NR)	Cycling	1.7%	150% bd loss	Hot	Normal	NA

Reis Silva *et. al* (2011)	Soccer	80	1200	RT	Normal<br>Above	NR
Ching Siow et al (2017)	NA	-	1825	RT	Normal	NA
J. SPACCAROTELLA *et. al* (2011)	Soccer	ex	-	NR	NR	Pre-season
Stanley *et. al* (2010)	Cycling	75	1000 + 12,5 ml por bd	Hot	Normal	NR
MF Sun *et. al* (2008)	Paddle	60<br>ex	free	RT	Below<br>Above	Pre-season
A. Tucker *et. al* (2015)	NA	3%	75 ml por bd	NA	NA	NA
VALIENTE *et. al* (2009)	Wrestling	120	150% bd loss	NR	NR	NR
VRIJENS *et. al* (1999)	Cycling	180	bd loss	Hot	Above	NR
Watson P *et. al* (2012)	Cycling	ex	-	Cool<br>RT	Normal	NA
WILKAN *et al.* (1996)	Cycling	80	free	Hot	Normal	NA
Wilk *et. al* (1998)	Cycling	60	free	Hot	Normal	NR
Wilk *et. al* (2007)	Cycling	80	free	Hot	Normal	NA
E. Wing *et al.* (2004)	Mountain bikers	48 km	2.8% bd<br>3600	RT	Normal	NR
Yanagisawa *et al.* (2012)	Skiing	-	3500	NR	NR	NR

Yun *et al.* (2022)	NA	-	3050	NA	NA	NR
BACKHOUSE *et al.* (2005)	Cycling	120	26 ml por bd	NR	NR	NR
Soon Gi Baek *et. al* (NR)	Treadmill	60	-	Hot	Above	NA
J. Baguley *et. al* (2016)	Cycling<br>Treadmill	2%	free	RT	Normal	NA
P. Bailey *et al.* (2008)	Cycling	ex	-	Hot<br>RT	Normal<br>Above	NA
Barr SI *et al.* (1991)	Cycling	360	bd loss	RT	Normal	NR
J. BATY *et al.* (2007)	Resistence training	3 sets 8 reps	1064	NR	NR	NA
D. Blacker *et al.* (2011)	Treadmill	120	900	RT	NR	NA
E. Bradbury *et al.* (2020)	Treadmill	80<br>3,22 km	-	NR	NR	NA
Capitán-Jiménez *et al.* (2022)	Cycling<br>Treadmill	60<br>4%	free	Hot	Above	NA
Carter JE *et al.* (1989)	Cycling	180	free	Hot	Below	NA
M. Christensen *et al.* (2012)	Cycling	14<br>146	500 + 12 ml por bd + free	RT	Below	Competitive phase
J. Clapp *et. al* (2000)	Endurance training	120	free	Hot	NR	NA
JS Costa *et al.* (2013)	Running	-	free	Hot	Normal	NA

Criswell *et al.* (1992)	Cycling	-	675	RT	Normal	NA
Currell *et al.* (2009)	Football	90	1100	NR	NR	NR
Currell *et al.* (2008)	Cycling	120	2250	RT	Normal	NA
Davis *et al.* (1990)	NA	-	275	NR	NR	NA
M. Davis *et al.* (1988)	Cycling	ex	-	RT	Above	NR
Mark Davis *et al.* (1997)	Cycling	ex	-	NR	NR	NA
Mark Davis *et al.* (1998)	Cycling	120	-	RT	Above	NR
M. Davis *et al.* (1999)	Running	75 min ex	7 ml por bd	NR	NR	NA
Davison *et al.* (2008)	Running	60 ex	8 ml por bd	NR	NR	NA
Del coso *et al.* (2008)	Cycling	120	sweat loss	Hot	Below	NA
Espino-González *et al.* (2018)	Cycling	37,2 km	-	RT	Below Normal	NR
D. Fahey *et al.* (1991)	Cycling	540	7000	NR	NR	NR
Weiping Fan *et al.* (2020)	Cycling	120 20 km	150% sweat loss	RT	Below Normal	NA
MARK A *et al.* (1996)	Cycling	ex	-	Hot	Below Normal	NR

Fernández-Campos *et al.* (2015)	Volleyball	-	6 ml por bd	NR	NR	NR
W. Glace *et al.* (2018)	Cycling	120<br>3 km	-	NR	NR	NR
GLICKMAN-WEISS *et al.* (1995)	NA	-	1892	RT	Above	NA
Goh *et al.* (2012)	Cycling	130	-	RT	NR	NR
R. Goldstein *et al.* (2023)	Cycling	22<br>ex	1000	NR	NR	NR
Harper *et al.* (2015)	Soccer	120	700	RT	Above	NR
Harper *et al.* (2017)	Soccer	90	500 + free	RT	Normal	NR
S. Hickey *et. al* (NR)	Running	120	free	Hot	Normal	NR
HoRIE *et al.* (2003)	NA	-	free	Hot<br>RT	NR	NA
Pryor *et al.* (NR)	Running	140	free	NR	NR	NR
Kamijo *et al.* (2012)	Cycling	2,30%	bd loss	Hot	Normal	NA
Keen *et al.* (2016)	Cycling	3%	bd loss	RT	NR	NR
Klimešová *et al.* (2019)	NA	-	-	NA	NA	NR
Koulmann *et al.* (1997)	Treadmill	60<br>2%	50% bd loss	Hot	Below	NA

Lambert *et al.* (1992)	Cycling	4,12%	bd loss	Hot	Normal	NA
Lambert *et al.* (NR)	Running	120	15 ml por bd	Hot	Below	NR
Lyons *et al.* (1990)	Treadmill	90	28.4 ml por bd<br>5.4 ml por bd	Hot	Below	NA
Maughan *et al.* (1994)	Cycling	1,80%	bd loss	Hot	Above	NA
A. McRae *et al.* (NR)	Tennis	120	23 ml por bd	RT	Normal	NR
Millard-Stafford *et al.* (NR)	Running	15 km	1000 + free	RT	Above	NR
Millard-Stafford *et al.* (2010)	Swimming	5 sets 200 reps	12 ml por bd	NR	NR	post-season
Millard-Stafford *et al.* (1992)	Running	40 km	2400	Hot<br>RT	Above	NR
MITCHELL *et al.* (2000)	Running	10 km	2475	Hot<br>RT	Above	Competitive phase
Mitchell JB *et al.* (2016)	Cycling	90<br>2,5%	100% bd loss<br>150% bd loss	Hot<br>RT	Normal	NA
Molaeikhaletabadi *et al.* (2022)	Badminton	90	3500	RT	NR	NR
Moreno *et al.* (2013)	Treadmill	90	bd loss	RT	Normal	NA
Morito *et al.* (2022)	Cycling	60	1050	RT	Normal	NR
Murray *et al.* (1989)	Cycling	-	100 ml por bd	Hot	Normal	NA

Naito *et al.* (2022)	NA	-	6,25 ml por bd	Hot	Normal	NA
Nakamura *et al.* (2021)	Treadmill	ex	4 ml por bd	Hot	Above	NR
Newell *et al.* (2015)	Cycling	120	2000	RT	NR	NR
Jason *et al.* (2023)	Cycling	75	free	Hot	Normal	NA
Jason *et al.* (2018)	Walking	4 km<br>30 min	1186,7	Hot	Normal	NA
NILES *et al.* (2001)	Running	ex	1200	RT	NR	NR
O'Reilly *et al.* (2013)	Soccer	75	8 ml por bd	Hot	Above	NR
Otskua *et al.* (2021)	Cycling	17	-	RT	Normal	NR
Owen MD *et al.* (1986)	Running	120	1200	Hot<br>RT	NR	NR
Palmer *et al.* (2017)	Hockey	90	sweat loss	RT	Below	NR
Papacosta *et al.* (2015)	Judo	900	1000	NA	NA	Pre-season
Park *et al.* (2012)	Treadmill	2%	free	Hot	Normal	NA
Peart *et al.* (2016)	Cycling	60	750	NR	NR	NA
Peschek *et al.* (2014)	Running	30<br>5 km	-	NR	NR	NR

Powers *et al.* (1990)	Cycling	ex	-	RT	Normal	NR
Pross *et al.* (2013)	NA	-	free	NA	NA	NA
Rollo *et al.* (2012)	Running	16 km	free<br>2000	RT	Above	NR
Rowlands (2011)	Cycling	120	2000	RT	Normal	NR
Rowlands (2012)	Mountain bikers	150<br>94	-	RT	Normal	Competitive phase
Rutherford *et al.* (2010)	Cycling	90	500	RT	NR	NR
Ryan *et al.* (1991)	Treadmill	60<br>120	1000<br>free	Hot	Normal	NA
Aoki *et al.* (2003)	Endurance training	-	1000	NR	NR	NA
Sanders *et al.* (1999)	Cycling	90	1200	Hot	Normal	NR
Schleh *et al.* (2018)	Walking	90	150% sweat loss	Hot	Normal	NA
DS *et al.* (1991)	Walking	30 km	free<br>6000	RT	Normal	NA
Shirreffs *et al.* (2007)	NR	2%	150% bd loss	Hot	Above	NA
Skillen *et al.* (2008)	Cycling	90<br>ex	24,8 ml por bd + 500	RT	Normal	NR
Smith *et al.* (2017)	Endurance training	3 sets 6 reps	948	NR	NR	NA

Smith *et al.* (2017)	Endurance training	5 sets ex	708	NR	NR	NA
Snell wt al. (2010)	Treadmill	60<br>ex	bd loss	Hot<br>RT	Below	NR
Matt S. *et al.* (2010)	Endurance training	6 sets ex	-	NR	NR	NR
Takada *et al.* (2022)	Running	90	1000	Hot	Normal	NR
Trong *et al.* (2015)	Cycling	27,5 km	2090	Hot	Normal	NR
Upshaw *et al.* (2016)	Cycling	ex<br>20 km	-	NR	NR	NR
Utter AC *et al.* (2002)	Running	-	-	NR	NR	NR
Wilkerson *et al.* (2012)	Cycling	80,5 km	500	NR	NR	NR
Wilson *et al.* (2016)	Walking	120<br>6,4 km	-	RT	Below	NR
WONG *et al.* (1997)	Treadmill	90 min<br>ex	free<br>bd loss	RT	Normal	NR
El-Sayed *et al.* (1996)	Cycling	60	312	RT	Normal	NR
FEBBRAIO *et al.* (2000)	Cycling	120	free	NR	NR	NR
Ferguson-Stegall *et al.* (2010)	Cycling	180<br>ex	-	RT	NR	NR
Finn *et al.* (2004)	Wrestling	-	-	NR	NR	Pre-season

Flood *et al.* (2020)	Cycling	105	1001	Hot	Below	NA
F Gilson *et al.* (2010)	Soccer	930	-	NR	NR	Post-season
Green *et al.* (2008)	Running	30	-	NR	NR	NA
Luden *et al.* (2007)	Running	90 km<br>93 km<br>60	-	NR	NR	NR
Onitsuka *et al.* (2018)	NA	-	7.5 ml por bd	RT	Normal	NA
Palmer *et al.* (1998)	Cycling	20 km	8 ml por bd	NR	NR	NR
Price *et al.* (2012)	Running	48	4,5 ml por bd	NR	NR	NA
ALAN C *et al.* (2005)	Endurance training	4 sets 10 reps	18 ml por bd	NR	NR	NR
Utter AC *et al.* (2004)	Walking	180	16 ml por bd	RT	Below<br>Normal	NR
C. utter *et al.* (1999)	Running<br>Cycling	150	24 ml por bd<br>52 ml por bd	RT	Below<br>Normal	NR
J. Valentine *et al.* (2008)	Cycling	ex	-	RT	NR	NR
Warber *et al.* (2000)	Treadmill<br>Running	240<br>ex	-	NR	NR	NR
WIDRICK *et al.* (1993)	Cycling	70 km	16,45 ml por bd	RT	Normal	NR
WILLIAMS *et al.* (2003)	Cycling	120	710	RT	NR	NR

Wojcik *et al.* (2001)	Endurance training	10 sets 10 reps	-	NR	NR	NA

Legend: **Green** stands for studies applying an inter-set intake strategy, **blue** for post-set, **yellow** for pre-set, **orange** for pre-set and inter-set, **brown** for pre-set and post-set, **gray** for inter-set and post-set, **dark green** for pre-set, inter-set, and post-set, and **dark blue** for non-applicable. % bd - % body weight, % bd loss - % body weight loss, % Sweat loss - % sweat weight loss, Bd loss – body weight loss, COOL – cool environment, Ex – until exhaustion, Free - ad libitum, HOT – hot environment, Km – kilometers, Min – minutes, mL per body – milliliters per body weight, mL – milliliters, NA – non-applicable, NR – non-reported, Reps – repetitions, RT – room temperature.

Reporting details: Studies that were not clear regarding their information of exercise or intake protocol were filled with "–". When authors provided information of intake protocol such as "subjects drank *X* mL of fluid per *Y* min of exercise", we tried our best to infer the correct quantity of fluid taken considering the information provided for exercise protocol. The duration of exercise and quantities of fluid taken herein presented refer to the total amount. If such value was provided by authors, a counting of timings or drinking was made. 1 L was considered equivalent to 1 kg. Temperate and relative humidity were qualitatively evaluated based on and https://www.nationalasthma.org.au/news/2016/indoor-humidity, despite how authors described it. Dry bulb temperature was always considered over wet bulb temperature. Conditions Below/Above, Normal/Above and Below/Normal result from studies where two interventions were performed under different relative humidities or from articles where relative humidity ranged between two conditions.
