# Supplementary material for: Trends and Missing Links in (De)Hydration Research: A Narrative Review
Source: Nutrients. 2024 May 30;16(11):1709. doi: 10.3390/nu16111709 (PMC11174495; doi:10.3390/nu16111709)
Supplement: Supplementary file 1 [file nutrients-16-01709-s001.zip › OSF4 Table S4 - Beverages and outcomes details.pdf]

Supplementary Table S4. Beverages and outcomes details

Study (year)	Beverage type	Beverage composition	Performance outcomes	Health outcomes	Hydration outcomes
Abbey *et.al.* (2009)	Honey<br>Sports drink<br>Unknown placebo	NR	Performance skill<br>Time to exhaustion<br>RPE	Blood measures	NA
Alexy *et al.* (2011)	NA	NA	NA	NA	urine ouput<br>Urine osmolality<br>Urine electrolytes<br>Free water
Ali *et al.* (2010)	CHO-E<br>Water	CHO<br>Sugars<br>Sodium<br>Potassium<br>Others	RPE	Heart rate	Body weight changes
Amano *et al.* (2019)	Water<br>CHO	CHO<br>Sodium<br>Calcium<br>Magnesium<br>Potassium<br>Others	Performance time	Blood measures<br>Hct/Hb<br>Temperature<br>Vital signs	Body weight changes<br>Fluid intake<br>Plasma volume changes<br>Plasma osmolality<br>Sweat rate outputss<br>Urine outputs<br>Cell volume

Amano *et al.* (2022)	CHO-E<br>Water	CHO<br>Sugars<br>Sodium<br>Calcium<br>Magnesium<br>Potassium	Respiratory ouputs	Blood measures<br>Subjective feelings	Urine outputs<br>BHI<br>Plasma volume changes<br>Cell volume<br>Plasma osmolality
Anastasiou *et al.* (2009)	Gatorade<br>Water	CHO<br>Potassium<br>Sodium	Muscle damage	Blood measures	Plasma volume changes<br>Plasma osmolality<br>Plasma electrolytes<br>Aldosterone
Armstrong *et al.* (2010)	NA	NA	NA	NA	Body weight changes<br>Plasma osmolality<br>Fluid intake<br>Urine outputs<br>USG<br>Urine color<br>Urine creatinine excretion<br>Blood urea
Arnaoutis *et al.* (2012)	Water	Sodium<br>Water	Time to exhaustion<br>Performance time<br>Total work<br>RPE	Blood measures<br>Heart rate	Dehydration %<br>Plasma volume changes

Bachle *et al.* (1998)	Water<br>gatorade	NR	Power output<br>RPE	Heart rate	Body weight changes<br>Urine outputs<br>Urine osmolality<br>Urine electrolytes<br>Fluid intake<br>Sweat outputs
Bechke *et al.* (2022)	Water<br>Others	CHO<br>Sodium<br>Potassium	NA	Vital signs<br>Heart rate	BHI<br>Net fluid balance<br>Urine outputs<br>Total body water<br>Intracellular water<br>Extracellular water
Berry *et al.* (2020)	Water<br>CHO-E<br>milk	CHO<br>Sodium<br>Potassium	NA	Blood measures	Urine outputs<br>Net fluid balance<br>BHI<br>Plasma osmolality<br>Urine osmolality<br>Plasma electrolytes<br>Urine electrolytes<br>Free water<br>Plasma volume changes
Brandenburg *et al.* (2012)	Water<br>Sports drink	NR	Performance time<br>Performance skill	NA	USG<br>Fluid loss<br>Body weight changes<br>Sweat outputs<br>Fluid intake

Briars *et al.* (2017)	Sports drink<br>Water	CHO<br>Sugars<br>Sodium	Performance time	NA	Dehydration %
Mehmet Cebi (2017)	Sports drink<br>Water	NA	NA	Blood measures	Body weight changes<br>Fluid intake
Chia *et al.* (2011)	Sports drink<br>Water<br>Others	Sodium<br>CHO<br>Water	NA	NA	Body weight changes<br>USG
Clarke *et al.* (2019)	Water<br>gatorade<br>Sports drink	CHO<br>Sodium<br>Potassium<br>AA	NA	NA	Urine outputs<br>BHI<br>Net fluid balance<br>eGFR<br>Plasma volume changes
Davies *et al.* (2023)	Water<br>Sports drink	Sodium	NA	NA	Plasma osmolality<br>Plasma volume changes<br>Body weight changes<br>Net fluid balance
Vieira de Carvalho *et al.* (2007)	Water<br>CHO-E	CHO<br>Sugars<br>Sodium<br>Potassium<br>Chloride	Lactic acid	Blood measures<br>Hct/Hb<br>Blood measures<br>Blood cells count	Body weight changes<br>Urine electrolytes<br>Urine color<br>USG
Demirhan *et al.* (2017)	Water<br>Sports drink	NR	NA	Temperature	Body weight changes<br>Plasma electrolytes
Desbrow *et al.* (2014)	milk<br>Sports drink	Protein<br>Others<br>CHO<br>Sodium	NA	Blood measures<br>Hct/Hb<br>Taste perceptions<br>Subjective feelings	Urine electrolytes<br>Plasma volume changes<br>Urine outputs<br>Body weight changes<br>Fluid retention

García-Berger *et al.* (2020)	gatorade<br>Sports drink	Protein<br>Others<br>CHO<br>Sodium	Performance time	NA	Body weight changes<br>USG<br>Urine color
D.B. Goulet *et al.* (2008)	Water<br>Sports drink<br>glycerol beverage	NR	Power output<br>Time to exhaustion<br>RPE	Heart rate<br>Temperature<br>Subjective feelings	Body weight changes<br>Urine outputs
R. Harris *et al.* (2019)	Water<br>gatorade	Sodium<br>Chloride<br>Potassium<br>Magnesium<br>Calcium<br>Others<br>CHO	Torque<br>Performance skill	Heart rate<br>Temperature	Saliva osmolality<br>Body weight changes<br>Sweat outputs<br>Time to Dehydration % (3%)
Heileson *et al.* (2022)	sport drink<br>CHO	CHO<br>Sugars<br>Sodium<br>Potassium	RPE	Subjective feelings	USG<br>Body weight changes
J. Hill *et al.* (2008)	Sports drink<br>Water	CHO<br>Sugars<br>Protein<br>Others<br>Sodium<br>Potassium<br>Magnesium	NA	NA	Fluid retention<br>Fluid intake

I Ismail *et al.* (2007)	Otherss<br>E<br>Sports drink<br>Water	CHO<br>Sugars<br>Sodium<br>Potassium<br>Chloride	NA	Hct/Hb<br>Blood measures<br>Taste perceptions<br>Subjective feelings	Body weight changes<br>Urine electrolytes<br>Plasma osmolality<br>Urine outputs<br>Urine osmolality<br>Plasma volume changes<br>Net fluid balance<br>% Rehydration %
S Kalman *et al.* (2012)	Water<br>Others<br>Sports drink	NR	Performance time	Subjective feelings<br>Heart rate<br>Vital signs	Body weight changes<br>Fluid retention<br>Plasma osmolality<br>USG<br>Rehydration %<br>Fluid retention
Malisova *et al.* (2016)		NA	NA	Hct/Hb	Urine color<br>Urine osmolality<br>Plasma osmolality<br>Urine electrolytes<br>Plasma electrolytes<br>USG<br>Urine outputs
Kitson *et al.* (2021)	ORS<br>sport drink<br>Water	Sugars<br>CHO<br>Sodium<br>Potassium<br>Others<br>Chloride	RPE<br>Felt arousal scale	Taste perceptions<br>Thermal comfort<br>Subjective feelings<br>Temperature<br>Heart rate	Urine osmolality<br>Saliva osmolality<br>USG<br>Urine color<br>Body weight changes

Kurdak *et al.* (2010)	Water<br>Sports drink	NR	NA	Temperature	Sweat outputs<br>Body weight changes<br>Sweat electrolytes<br>Urine outputs<br>USG
K. W. Lee *et al.* (2011)	Water<br>Sports drink<br>Unknown placebo	Others<br>Protein<br>CHO<br>Sugars<br>Sodium<br>Potassium	RPE<br>Time to exhaustion	Taste perceptions<br>Subjective feelings<br>Temperature<br>Thermal comfort<br>Heart rate<br>Hct/Hb<br>Blood measures	Sweat outputs<br>Fluid intake<br>Urine outputs<br>Fluid retention<br>Body weight changes<br>Plasma volume changes<br>Urine osmolality
Matias *et al.* (2019)	Others<br>Water	CHO<br>Calcium<br>Magnesium<br>Others<br>Potassium<br>Sodium<br>Chloride	NA	Subjective feelings<br>Heart rate	Body weight changes<br>Urine outputs<br>Urine osmolality<br>USG<br>Urine color<br>Fluid loss<br>Saliva osmolality<br>Fluid retention<br>Urine antioxidant proprerties

J. Maughan *et al.* (2007)	Sports drink<br>Water	CHO<br>Sodium<br>Protein	NA	NA	Urine outputs<br>Sweat outputs<br>Body weight changes<br>Urine osmolality<br>Sweat electrolytes<br>Fluid intake
MCBRIDE *et al.* (2020)	CHO-PRO	CHO<br>Protein<br>Sodium<br>Potassium	NA	Subjective feelings	Fluid retention<br>Hydration %<br>Fluid loss<br>Urine outputs
Meyer *et al.* (1994)	Others<br>Water	CHO<br>Sodium<br>Potassium	NA	Temperature<br>Heart rate<br>Taste perceptions<br>Subjective feelings	Rehydration %<br>Body weight changes<br>Sweat outputs<br>Urine outputs
Millard-Stafford *et al.* (1995)	CHO-E	CHO<br>Sugars<br>Sodium<br>Chloride<br>Potassium	Vo2<br>Performance time	Blood measures<br>Hct/Hb<br>Heart rate<br>Temperature	Plasma volume changes<br>Plasma osmolality<br>Body weight changes<br>Sweat outputs<br>Fluid intake<br>Urine outputs<br>Respiratory loss<br>Plasma electrolytes
L. Millard-Stafford *et al.* (2005)	powerade<br>gatorade<br>sweet placebo	CHO<br>Sugars	RPE<br>Substrate utilization<br>Vo2<br>Respiratory ouputs<br>Performance time<br>Lactic acid	Heart rate<br>Temperature<br>Taste perceptions<br>Hct/Hb<br>Blood measures	Body weight changes<br>Plasma electrolytes<br>Plasma volume changes<br>Fluid retention<br>Plasma osmolality

MITCHELL *et al.* (2000)	E	Sodium	Vo2	Vital signs<br>Heart rate<br>Hct/Hb	Plasma osmolality<br>Plasma electrolytes<br>Urine electrolytes<br>% Dehydration<br>% Rehydration<br>Plasma volume changes<br>Sweat outputs<br>Urine outputs<br>Plasma ADH
Mora-Rodriguez *et al.* (2016)	NA	NA	Performance skill	Hct/Hb<br>Blood measures	Urine outputs<br>Urine osmolality<br>Plasma electrolytes<br>Urine electrolytes<br>Non-renal water loss<br>Free water<br>Fluid intake
K. O'Neal *et al.* (2014)	Water<br>Others<br>Sports drink	NR	Performance time<br>Performance skill	Heart rate	USG<br>Sweat outputs<br>Body weight changes<br>Fluid intake<br>Urine outputs
K. O'Neal *et al.* (2012)	Water<br>E<br>sweet<br>E-sweet	Sodium<br>Potassium<br>Chloride<br>Others	Performance time<br>Performance skill<br>RPE	Heart rate<br>Taste perceptions<br>Subjective feelings	USG<br>Sweat outputs<br>Fluid intake<br>Urine outputs

L. Osterberg *et al.* (2009)	Water<br>Sports drink	CHO<br>Sodium	NA	NA	USG<br>Fluid intake<br>Sweat outputs<br>Body weight changes<br>Sweat electrolytes
Luke Pryor *et al.* (2012)	CHO-E<br>CHO-E-betaine	Others	Power output	NA	NA
Ramos-Jiménez *et al.* (2013)	Water<br>gatorade	CHO<br>Sodium<br>Potassium	Performance skill	Temperature<br>Heart rate<br>Vital signs	Body weight changes
M. RIVERA-BROWN *et al.* (1999)	Water<br>CHO-E	CHO<br>Sodium	RPE<br>Total work<br>Vo2	Metabolic heat<br>Production/storage<br>Thermal comfort<br>Subjective feelings<br>Temperature<br>Hct/Hb<br>Heart rate	Body weight changes<br>Fluid intake<br>Sweat outputs<br>Respiratory loss<br>Fluid loss<br>Urine outputs<br>Plasma electrolytes<br>Plasma volume changes<br>Plasma osmolality

M. Rivera-Brown *et al.* (2008)	Water<br>CHO-E	CHO<br>Sodium	RPE<br>Total work<br>Vo2	Metabolic heat<br>Production/storage<br>Thermal comfort<br>Subjective feelings<br>Temperature<br>Hct/Hb<br>Heart rate	Body weight changes<br>Fluid intake<br>Sweat outputs<br>Respiratory loss<br>Fluid loss<br>Urine outputs<br>Plasma electrolytes<br>Plasma volume changes<br>Plasma osmolality
D Roberts *et al.* (2014)	CHO<br>sweet-placebo	CHO<br>Sugars<br>Protein<br>Others<br>Sodium	Vo2<br>VCo2<br>Respiratory ouputs<br>RPE<br>Performance skill<br>Power output<br>Performance time<br>Performance skill<br>Substrate utilization<br>Expired gas13C:12C ratio	Heart rate<br>Subjective feelings<br>2h2o<br>Blood measures	NA
Rodriguez-Giustiniani *et al.* (2018 accepted)	gatorade<br>E-placebo	Sodium<br>CHO<br>Sugars	Performance skill	Subjective feelings<br>Blood measures	Body weight changes<br>Fluid intake<br>Urine outputs<br>Urine osmolality

Rollo *et al.* (2021)	Water<br>CHO-E	NR	RPE<br>Performance skill	NA	USG<br>Body weight changes<br>Sweat outputs<br>Urine outputs<br>Fluid intake<br>Sweat electrolytes
J. Saunders *et al.* (2004)	CHO-PRO<br>gatorade	CHO<br>Protein	Time to exhaustion<br>Performance skill<br>Creatine kinase levels<br>RPE<br>Lactic acid<br>Vo2<br>VCo2<br>Respiratory ouputs	Heart rate<br>Blood measures	NA
Schrader *et al.* (2016)	Water<br>CHO	CHO<br>Sugars	Respiratory ouputs	Hct/Hb<br>Blood measures<br>Vital signs	Plasma electrolytes<br>Plasma osmolality<br>Plasma volume changes<br>Urine outputs<br>Body weight changes<br>Total body water<br>Extracellular water<br>Intracellular water
G. Schweitzer *et al.* (NR)	gatorade<br>Unknown placebo	NR	Vo2<br>RPE<br>Total work<br>Performance skill	Blood measures<br>Heart rate	NA

M. Shirreffs *et al.* (NR)	milk<br>Water<br>Sports drink<br>E	CHO<br>Others<br>Protein<br>Sodium<br>Potassium<br>Chloride	NA	Taste perceptions<br>Subjective feelings	Sweat outputs<br>Body weight changes
Reis Silva *et al.* (2011)	CHO-E<br>Others	CHO<br>Sugars<br>Others	Performance skill	Heart rate	USG<br>Body weight changes<br>% Dehydration<br>Sweat outputs
Ching Siow *et al.* (2017)	Water<br>Others	Sodium<br>Potassium<br>Chloride<br>CHO<br>Sugars<br>Protein<br>Others	NA	Hct/Hb	Urine outputs<br>Plasma osmolality<br>Urine osmolality<br>Saliva osmolality<br>Plasma electrolytes<br>Urine electrolytes<br>USG<br>Saliva output<br>Fluid retention<br>Fluid intake<br>Free water reserve<br>Urine color
J. Spaccarotella *et al.* (2011)	milk<br>gatorade	Protein<br>Others<br>CHO<br>Calcium<br>Sodium	RPE<br>Time to exhaustion	NA	NA

Stanley *et al.* (2010)	powerade	CHO<br>Sodium	RPE<br>Power output	blood pH ysiological strain index<br>Hct/Hb<br>Blood measures<br>Heart rate<br>Temperature<br>Thermal comfort	Body weight changes<br>Plasma volume changes<br>USG<br>Sweat outputs<br>Plasma electrolytes<br>Cell volume
MF Sun *et al.* (2008)	gatorade<br>Water	CHO<br>Sugars<br>Sodium<br>Potassium	RPE<br>Performance skill	Temperature<br>Subjective feelings	Urine outputs<br>USG<br>Fluid intake<br>Fluid loss<br>% Dehydration
A. Tucker *et al.* (2015)	Water<br>Others	NR	Creatine kinase levels	Hct/Hb<br>Blood measures	Body weight changes<br>Total body water<br>Intracellular water<br>Extracellular water<br>Urine color<br>USG<br>urea nitrogen<br>Urine outputs<br>Free water<br>Plasma osmolality<br>Plasma electrolytes<br>Urine electrolytes<br>Urine osmolality

VALIENTE *et al.* (2009)	Water<br>gatorade	Sodium<br>Potassium<br>CHO	NA	Hct/Hb	USG<br>Urine osmolality<br>Plasma osmolality<br>Plasma volume changes
VRIJENS *et al.* (1999)	Water<br>E	Sodium<br>CHO<br>Potassium	RPE	Heart rate<br>Temperature<br>Hct/Hb<br>Blood measures	Urine electrolytes<br>Body weight changes<br>Urine outputs<br>Plasma electrolytes<br>Plasma Aldosterone<br>Sweat outputs<br>Fluid intake
Watson P *et al.* (2012)	Others	CHO<br>Sugars<br>Sodium<br>Potassium	Energy expenditure<br>RPE<br>Vo2<br>Substrate utilization	Heart rate<br>Temperature<br>Thermal comfort	Body weight changes<br>Sweat outputs
Wilkan *et al.* (1996)	Water<br>CHO	CHO<br>Sugars<br>Others	NA	Temperature<br>Heart rate<br>Subjective feelings	% Hydration<br>Body weight changes<br>Urine outputs<br>Sweat outputs<br>Fluid intake<br>Respiratory loss
Wilk *et al.* (1998)	CHO	CHO<br>Sugars<br>Others	NA	Temperature<br>Heart rate<br>Subjective feelings	% Hydration<br>Body weight changes<br>Urine outputs<br>Sweat outputs<br>Fluid intake<br>Respiratory loss

Wilk *et al.* (2007)	Water<br>CHO	CHO<br>Sugars<br>Others	NA	Temperature<br>Heart rate<br>Subjective feelings	% Hydration<br>Body weight changes<br>Urine outputs<br>Sweat outputs<br>Fluid intake<br>Respiratory loss
E. Wing *et al.* (2004)	Water<br>Water-glycerol	Others<br>Water	RPE	Subjective feelings<br>Thermal comfort<br>Heart rate<br>Temperature<br>Blood measures	body mas<br>Urine outputs<br>Urine color<br>USG<br>Fluid intake<br>Sweat outputs
Yanagisawa *et al.* (2012)	Water<br>CHO-E	Sodium<br>Potassium<br>CHO	Vo2	Hct/Hb<br>Heart rate	Body weight changes<br>Plasma electrolytes<br>Plasma osmolalitytic pressure<br>Urine outputs<br>plasma Aldosterone<br>USG<br>Urine electrolytes<br>Plasma volume changes
Yun *et al.* (2022)	Water<br>ORS<br>Sports drink	CHO<br>Others<br>Protein<br>Sodium<br>Potassium<br>Chloride	NA	Hct/Hb	Fluid intake<br>Body weight changes<br>Urine outputs<br>BHI<br>Plasma volume changes

Backhouse *et al.* (2005)	Water<br>CHO-E	NR	Vo2<br>VCo2<br>RPE	Blood measures<br>Heart rate<br>Taste perceptions	NA
Soon Gi Baek *et al.* (NR)	Water<br>E<br>Others	NR	NA	Blood measures	NA
J. Baguley *et al.* (2016)	powerade<br>milk	Protein<br>Others<br>CHO<br>Sodium	NA	Subjective feelings<br>Taste perceptions	USG<br>Body weight changes<br>Fluid intake<br>Urine outputs<br>Net fluid balance<br>Fluid retention
P. Bailey *et al.* (2008)	CHO<br>Water	CHO<br>Sodium<br>Potassium	Time to exhaustion<br>Performance skill	Heart rate<br>Temperature	Body weight changes
Barr SI *et al.* (1991)	Water<br>E	Others	RPE<br>Vo2<br>VCo2<br>Respiratory ouputs	Heart rate<br>Temperature<br>Hct/Hb	Body weight changes<br>Plasma electrolytes<br>Urine electrolytes<br>Sweat electrolytes<br>Plasma Aldosterone<br>Plasma volume changes<br>Fluid intake<br>Sweat outputs<br>Urine outputs<br>Respiratory loss<br>Fluid retention

J. BATY *et al.* (2007)	CHO-PRO<br>Unknown placebo	NR	Creatine kinase levels<br>Myoglobin concentration<br>Performance skill<br>Muscle damage	Blood measures<br>Subjective feelings	NA
D. Blacker *et al.* (2011)	Water<br>CHO-E	CHO<br>Protein<br>Others<br>Sugars<br>Water	Respiratory ouputs<br>Vo2<br>VCo2<br>RPE<br>Performance skill	Heart rate<br>Blood measures<br>Vital signs	Body weight changes<br>Urine outputs<br>Fluid intake
E. Bradbury *et al.* (2020)	CHO<br>unkown placebo	CHO<br>Sugars<br>Protein	Performance time<br>Vo2<br>RPE	Heart rate	NA
Capitán-Jiménez *et al.* (2022)	Water		NA	Subjective feelings	Urine osmolality<br>Plasma osmolality<br>Urine color<br>Fluid intake<br>USG<br>Net fluid balance

Carter JE *et al.* (1989)	CHO-E<br>Water	Sugars<br>CHO<br>Sodium<br>Calcium<br>Magnesium<br>Chloride	RPE<br>Vo2	Temperature<br>Heart rate<br>Blood measures<br>Hct/Hb	Sweat outputs<br>Body weight changes<br>Fluid intake<br>Plasma volume changes<br>urine pH<br>Fluid intake<br>Respiratory loss<br>Urine outputs<br>Urine electrolytes<br>Plasma electrolytes<br>Plasma osmolality
M. Christensen *et al.* (2012)	Others<br>Unknown placebo	Others	Vo2<br>Respiratory ouputs<br>Power output<br>RPE<br>Performance skill	Blood measures<br>Heart rate	NA
J. Clapp *et al.* (2000)	Water<br>Sports drink<br>Unknown placebo	Potassium<br>Sodium<br>CHO<br>Sugars	RPE	Temperature<br>Heart rate<br>Subjective feelings<br>Thermal comfort	Fluid intake<br>Body weight changes<br>Sweat outputs<br>Fluid loss<br>Body weight changes

JS Costa *et al.* (2013)	NA	NA	NA	Hct/Hb	Body weight changes<br>Urine osmolality<br>Plasma osmolality<br>Urine color<br>Urine osmolality/Plasma osmolality rate<br>Plasma electrolytes<br>Total body water<br>Extracellular water<br>Intracellular water<br>Plasma volume changes
Criswell *et al.* (1992)	Water<br>CHO-E	CHO<br>Sugars	Respiratory ouputs	Temperature<br>Blood measures<br>Heart rate<br>Hct/Hb	Plasma electrolytes<br>Plasma volume changes<br>Plasma osmolality<br>plasma Aldosterone
Currell *et al.* (2009)	CHO<br>unkown placebo	NR	Performance skill	NA	NA
Currell *et al.* (2008)	Water<br>CHO	CHO	RPE<br>Power output<br>Performance skill<br>Vo2<br>VCo2<br>Substrate utilization	Blood measures<br>Heart rate	NA
Davis *et al.* (1990)	CHO<br>Water	Sodium<br>Potassium<br>Others	NA	Blood measures<br>2h2o	NA

M. Davis *et al.* (1988)	CHO<br>Water	CHO<br>Sugars<br>Sodium<br>Potassium	Performance time<br>Vo2<br>Respiratory ouputs	Hct/Hb<br>Blood measures<br>Temperature<br>Subjective feelings<br>Taste perceptions<br>2h2o	Plasma volume changes<br>Sweat outputs<br>Body weight changes<br>Fluid intake<br>Respiratory loss<br>Metabolic loss
Mark Davis *et al.* (1997)	CHO<br>unkown placebo	NR	Time to exhaustion<br>RPE	Hct/Hb<br>Blood measures<br>Heart rate	Plasma volume changes
Mark Davis *et al.* (1998)	Water<br>CHO-E	CHO<br>Sugars<br>Sodium<br>Potassium	Vo2<br>Performance time<br>Respiratory ouputs	2h2o<br>Hct/Hb<br>Heart rate<br>Temperature<br>Blood measures	Plasma volume changes<br>Sweat outputs<br>Body weight changes<br>Fluid intake<br>Respiratory loss
M. Davis *et al.* (1999)	CHO<br>CHO-AA<br>Water	CHO<br>Sodium<br>AA<br>Potassium	Time to exhaustion	Hct/Hb<br>Blood measures<br>Heart rate	Plasma volume changes
Davison *et al.* (2008)	powerade<br>sweet Water	CHO<br>Others	Time to exhaustion	Blood measures<br>Hct/Hb<br>Heart rate	Plasma electrolytes<br>Cell volume<br>Plasma volume changes<br>USG<br>Body weight changes

Del coso *et al.* (2008)	Water<br>gatorade<br>powerade<br>Sports drink	CHO<br>Sodium<br>Magnesium<br>Calcium<br>Chloride<br>Others	Torque<br>Power output<br>Vo2<br>VCo2	Temperature<br>Hct/Hb<br>Subjective feelings<br>Taste perceptions	Urine outputs<br>Body weight changes<br>Fluid intake<br>Sweat outputs<br>Respiratory loss<br>Plasma volume changes<br>Plasma osmolality<br>Plasma electrolytes<br>Urine osmolality<br>Urine electrolytes<br>Sweat electrolytes
Espino-González *et al.* (2018)	CHO-PRO<br>Sports drink	CHO<br>Protein	Power output<br>Performance time<br>RPE	Hct/Hb	Body weight changes
D. Fahey *et al.* (1991)	CHO<br>sweet Water	Sodium<br>Others<br>CHO<br>Sugars<br>Water	RPE<br>Vo2<br>Respiratory ouputs	Blood measures<br>venous<br>Heart rate<br>Vital signs<br>Temperature	Plasma electrolytes

Weiping Fan *et al.* (2020)	Water<br>Sports drink<br>Others	Others<br>Protein<br>CHO<br>Sodium<br>Potassium	RPE<br>Performance time	Hct/Hb<br>Blood measures<br>Heart rate<br>Temperature<br>Thermal comfort<br>Subjective feelings<br>Taste perceptions	Sweat outputs<br>Fluid intake<br>Urine outputs<br>Body weight changes<br>Fluid retention<br>Plasma volume changes<br>Cell volume<br>Net fluid balance<br>Urine osmolality<br>Urine electrolytes<br>Plasma Aldosterone<br>Plasma ADH<br>Plasma electrolytes<br>Plasma osmolality
MARK A *et al.* (1996)	CHO<br>sweet placebo	CHO<br>Sugars<br>Others	Performance time<br>RPE<br>Vo2	Blood measures<br>Hct/Hb<br>Heart rate<br>Temperature	Plasma volume changes
Fernández-Campos *et al.* (2015)	energy drink<br>E<br>Others	CHO<br>Sugars<br>Protein<br>Sodium<br>Potassium<br>AA	Performance skill<br>Power output<br>Muscle damage	NA	NA

W. Glace *et al.* (2018)	Sports drink<br>E	CHO<br>Sugars<br>Potassium<br>Sodium	RPE<br>Vo2<br>Respiratory ouputs<br>Performance skill<br>Performance time<br>Time to exhaustion	Blood measures<br>Heart rate	Body weight changes
Glickman-Weiss *et al.* (1995)	CHO-E<br>E	Sodium<br>CHO<br>Sugars	RPE	Blood measures<br>Temperature<br>Hct/Hb	Plasma electrolytes<br>Plasma osmolality
Goh *et al.* (2012)	CHO<br>CHO-PRO	CHO<br>Sugars<br>Protein<br>Others<br>AA	Muscle damage<br>Energy expenditure<br>Creatine kinase levels<br>Torque<br>Performance time<br>Vo2<br>Respiratory ouputs<br>RPE	Heart rate<br>Blood measures	NA
R. Goldstein *et al.* (2023)	gatorade<br>CHO<br>CHO-P	CHO<br>Protein	Time to exhaustion	Heart rate	Body weight changes<br>Fluid loss
Harper *et al.* (2015)	CHO<br>unkown placebo	CHO<br>Sugars<br>Others<br>Protein	Performance skill<br>RPE	Blood measures<br>Subjective feelings	Plasma electrolytes<br>Urine osmolality<br>Plasma osmolality<br>Urine outputs<br>Body weight changes
Harper *et al.* (2017)	CHO-E<br>E<br>Water	Sodium<br>CHO<br>Sugars	Performance skill<br>RPE	Heart rate<br>Subjective feelings<br>Cognitive capacities<br>Blood measures	Urine outputs

S. Hickey *et al.* (NR)	CHO<br>Water	CHO<br>Sodium<br>Potassium	Vo2<br>Respiratory ouputs	Heart rate<br>Blood measures<br>Hct/Hb<br>Temperature<br>Taste perceptions<br>Subjective feelings	Fluid intake<br>Urine outputs<br>Urine electrolytes<br>Urine osmolality<br>Sweat outputs<br>Body weight changes<br>Respiratory loss<br>Plasma volume changes<br>Plasma electrolytes<br>Plasma osmolality
HoRIE *et al.* (2003)	CHO-E	Sodium<br>Potassium<br>CHO	NA	Temperature<br>Subjective feelings	Body weight changes<br>Fluid intake<br>Urine outputs<br>Fluid loss<br>Urine electrolytes
Pryor *et al.* (NR)	powerade<br>Others	CHO<br>Protein<br>Others<br>Sodium<br>Water	Performance time<br>Total work<br>Energy expenditure	Heart rate<br>Subjective feelings<br>Vital signs	Urine outputs<br>Urine electrolytes<br>Sweat electrolytes<br>Body weight changes<br>Fluid loss<br>USG

Kamijo *et al.* (2012)	CHO<br>E	CHO<br>Sugars<br>Sodium<br>Potassium<br>Chloride<br>Others	Vo2	Hct/Hb<br>Blood measures	Plasma volume changes<br>Plasma Aldosterone<br>Sweat electrolytes<br>Urine osmolality<br>Urine elecrtro<br>Net fluid balance<br>Change in renal Na absorption<br>eGFR<br>Plasma osmolality<br>Urine inulin<br>Fluid loss
Keen *et al.* (2016)	Water<br>Sports drink	NR	Torque	NA	Body weight changes<br>Saliva osmolality
Klimešová *et al.* (2019)			NA	Taste perceptions	USG
Koulmann *et al.* (1997)	Water<br>CHO-E<br>CHO	Others	Vo2<br>VCo2<br>Respiratory ouputs	Heart rate<br>Temperature<br>Hct/Hb	Urine outputs<br>Body weight changes<br>Sweat outputs<br>Respiratory loss<br>Metabolic loss<br>Plasma electrolytes<br>Urine electrolytes<br>Sweat electrolytes

Lambert *et al.* (1992)	CHO<br>E<br>CHO-E	CHO<br>Sugars<br>Sodium<br>Potassium	Lactic acid<br>Respiratory ouputs	Hct/Hb<br>Blood measures<br>Heart rate<br>Temperature<br>Subjective feelings	Plasma volume changes<br>Body weight changes<br>Urine outputs<br>Plasma osmolality
Lambert *et al.* (NR)	CHO-E<br>E	CHO<br>Sodium<br>Potassium	NA	Blood measures<br>Hct/Hb<br>Heart rate<br>Subjective feelings<br>Temperature	Plasma osmolality<br>Plasma volume changes<br>Gastric electrolytes<br>Fluid intake<br>Sweat outputs<br>Body weight changes<br>Urine outputs<br>Fluid retention<br>Gastric osmolality
Lyons *et al.* (1990)	Water-glycerol<br>Water	NR	NA	Blood measures<br>Heart rate<br>Hct/Hb	Plasma osmolality<br>Plasma electrolytes<br>Sweat outputs<br>Fluid intake<br>Urine outputs
Maughan *et al.* (1994)	CHO<br>CHO-E<br>E	CHO<br>Sugars<br>Potassium<br>Chloride<br>Sodium	NA	NA	Fluid retention<br>Fluid intake<br>Urine outputs<br>Urine osmolality<br>Plasma osmolality<br>Urine electrolytes<br>Plasma volume changes

A. McRae *et al.* (NR)	CHO-E<br>E	NR	Performance skill<br>RPE<br>Felt arousal scale	Blood measures<br>Heart rate<br>Subjective feelings	Urine outputs<br>Urine osmolality<br>Body weight changes<br>Sweat outputs
Millard-Stafford *et al.* (NR)	Water<br>CHO-E	Sodium<br>Potassium	Vo2<br>RPE<br>Performance time<br>Substrate utilization<br>Respiratory ouputs	Hct/Hb<br>Blood measures<br>Heart rate	Plasma volume changes<br>Sweat outputs<br>Fluid intake<br>Urine outputs<br>Plasma electrolytes<br>Respiratory loss
Millard-Stafford *et al.* (2010)	Sports drink<br>Unknown placebo	NR	Performance skill<br>RPE	Blood measures<br>Heart rate	NA
Millard-Stafford *et al.* (1992)	CHO-E<br>Unknown placebo	Sugars<br>CHO<br>Sodium<br>Potassium	RPE<br>Respiratory ouputs	Blood measures<br>Temperature<br>Heart rate<br>Hct/Hb	Sweat outputs<br>Plasma electrolytes<br>Plasma osmolality<br>Plasma volume changes<br>Fluid intake<br>Urea nitrogen<br>Urine outputs
MITCHELL *et al.* (2000)	Water<br>powerade<br>gatorade<br>CHO	CHO<br>Sugars<br>Others<br>Sodium	Vo2<br>Performance time<br>RPE<br>Substrate utilization	Hct/Hb<br>Blood measures<br>Pre-exercise glycemic response	Plasma volume changes<br>Body weight changes

Mitchell JB *et al.* (2016)	E	Sodium<br>Chloride<br>Potassium	NA	Hct/Hb	Plasma volume changes<br>Plasma electrolytes<br>Plasma Aldosterone<br>Gastric output<br>Renin activity<br>Urine electrolytes<br>Urine outputs<br>Plasma electrolytes<br>Plasma osmolality<br>Sweat outputs
Molaeikhaletabadi *et al.* (2022)	Water<br>milk	Others<br>Protein<br>CHO<br>Calcium	RPE<br>Performance skill<br>Power output<br>Muscle damage	NA	NA
Moreno *et al.* (2013)	gatorade	CHO<br>Sodium<br>Chloride<br>Potassium	NA	Temperature<br>Heart rate	Body weight changes<br>USG
Morito *et al.* (2022)	Water<br>CHO-E	CHO<br>Sodium<br>Potassium	Power output<br>RPE	Temperature<br>Metabolic heat<br>Production/storage<br>Heart rate<br>Subjective feelings	Body weight changes

Murray *et al.* (1989)	CHO<br>Water	Sodium<br>Potassium<br>CHO<br>Sugars	RPE<br>Performance time<br>Vo2<br>Respiratory ouputs	Hct/Hb<br>Blood measures<br>Heart rate<br>Temperature<br>Subjective feelings<br>Taste perceptions	Plasma volume changes<br>Plasma osmolality<br>Plasma electrolytes
Naito *et al.* (2022)	CHO	CHO<br>Sodium<br>Chloride	NA	Temperature<br>Vital signs<br>Thermal comfort<br>Blood measures	USG<br>Body weight changes
Nakamura *et al.* (2021)	Sports beverage	CHO<br>Sodium<br>Potassium	NA	Blood pH<br>Phsiological strain index<br>Temperature<br>Vital signs<br>Heart rate	Body weight changes
Newell *et al.* (2015)	CHO<br>Water	Sodium	Performance time<br>Power output<br>Performance skill	NA	NA
Jason *et al.* (2023)	unkown	NR	RPE<br>Total work<br>Power output	Heart rate<br>Temperature<br>Vital signs<br>Blood pH<br>Phsiological strain index<br>Hct/Hb<br>Blood measures	Sweat outputs<br>Body weight changes<br>Plasma volume changes<br>Fluid intake

Jason *et al.* (2018)	unkown	NR	Vo2<br>Total work<br>RPE<br>Energy expenditure<br>Respiratory ouputs	Temperature<br>Heart rate<br>Blood pH<br>Phsiological strain index<br>Thermal comfort<br>Metabolic heat Production/storage	Sweat outputs<br>Body weight changes<br>Plasma volume changes<br>USG
NILES *et al.* (2001)	CHO<br>CHO-PRO	Sugars<br>CHO<br>Protein	Performance time	Blood measures	NA
O'Reilly *et al.* (2013)	CHO-E<br>unkown placebo	NR	Performance time<br>Performance skill	Blood measures<br>Temperature<br>Heart rate	Body weight changes<br>Sweat outputs<br>Fluid intake<br>Urine outputs<br>USG
Otskua *et al.* (2021)	Water<br>CHO-E	CHO<br>Sodium<br>Calcium<br>Magnesium<br>Potassium	RPE<br>Power output	Hct/Hb<br>Blood measures<br>Temperature<br>Thermal comfort<br>Subjective feelings<br>Taste perceptions	Plasma osmolality<br>Plasma electrolytes<br>Plasma volume changes<br>Cell volume<br>Sweat outputs<br>Body weight changes<br>Urine outputs<br>USG
Owen MD *et al.* (1986)	CHO<br>CHO-polymer<br>sweet Water<br>Water	Sugars<br>CHO<br>Sodium<br>Potassium	Respiratory ouputs	Blood measures<br>Heart rate<br>Temperature	Sweat outputs<br>Plasma volume changes<br>Plasma electrolytes

Palmer *et al.* (2017)	CHO-E	NR	Phosphocreatine (PCr)<br>Energy expenditure<br>Creatine kinase levels<br>Muscle free glucose	Blood measures	USG<br>Sweat electrolytes<br>Sweat outputs
Papacosta *et al.* (2015)	milk<br>Water	CHO<br>Sugars<br>Protein<br>Others<br>Sodium<br>Calcium	Total work<br>Muscle damage<br>RPE	Heart rate<br>Blood measures<br>Subjective feelings	Saliva output<br>Body weight changes
Park *et al.* (2012)	Water<br>Sports drink	NR	Performance time	Hct/Hb<br>Vital signs<br>Heart rate<br>Temperature	Plasma electrolytes<br>Plasma osmolality<br>Urine outputs<br>USG<br>Fluid intake<br>Body weight changes<br>Fluid intake<br>Plasma volume changes
Peart *et al.* (2016)	Water<br>Otherss	Sodium<br>CHO<br>Potassium	RPE<br>Performance time	Blood measures<br>Heart rate<br>Taste perceptions<br>Subjective feelings	Body weight changes<br>USG<br>Sweat outputs<br>Fluid intake

Peschek *et al.* (2014)	CHO-PRO	CHO<br>Protein<br>Others	Muscle damage<br>Performance time<br>Creatine kinase levels<br>Torque	NA	NA
Powers *et al.* (1990)	Unknown placebo<br>CHO-E polymer<br>E	Sodium<br>CHO<br>Potassium<br>Chloride	NA	venous<br>Blood measures<br>Hct/Hb<br>Heart rate<br>Temperature	Plasma volume changes<br>Plasma electrolytes<br>Plasma osmolality
Pross *et al.* (2013)	Water	NR	NA	Subjective feelings<br>Heart rate<br>Cognitive capacities	USG<br>Urine color<br>Urine outputs<br>Plasma osmolality<br>Saliva osmolality
Rollo *et al.* (2012)	gatorade	CHO<br>Sugars<br>Sodium<br>Potassium<br>Chloride<br>Magnesium	Performance time<br>RPE<br>Vo2<br>Performance skill	Temperature<br>Subjective feelings<br>Heart rate<br>Blood measures	Fluid intake<br>USG<br>Body weight changes<br>% Dehydration

Rowlands (2011)	gatorade<br>Sports drink<br>sweet Water	CHO<br>Sodium<br>Water	RPE	Hct/Hb<br>Blood measures<br>2h2o<br>Heart rate<br>Subjective feelings<br>Blood measures cell	Plasma volume changes<br>Plasma electrolytes<br>Plasma osmolality<br>Urine outputs<br>Urine osmolality<br>Fluid retention
Rowlands (2012)	CHO	CHO<br>Sugars<br>Sodium<br>Chloride<br>Potassium<br>Others	Power output<br>RPE<br>Performance time<br>Time to exhaustion<br>Total work<br>Muscle damage	Subjective feelings	Body weight changes<br>USG<br>Urine osmolality<br>Urine color
Rutherford *et al.* (2010)	Otherss<br>Unknown placebo	Others	Performance time<br>RPE<br>Respiratory ouputs<br>Substrate utilization<br>VCo2<br>Vo2	Heart rate	NA
Ryan *et al.* (1991)	CHO	CHO<br>Sugars<br>Sodium<br>Potassium	RPE	Subjective feelings<br>Taste perceptions<br>Heart rate<br>Temperature	Sweat outputs<br>Body weight changes<br>Fluid intake<br>Urine outputs
Aoki *et al.* (2003)	CHO<br>sweet placebo	NR	Total work<br>Performance skill	Blood measures	NA

Sanders *et al.* (1999)	Water<br>E	Others<br>Water	NA	Hct/Hb<br>Heart rate	Plasma volume changes<br>Plasma electrolytes<br>Sweat electrolytes<br>Urine electrolytes<br>Fluid loss<br>Body weight changes<br>Fluid intake<br>Urine outputs<br>Intracellular water
Schleh *et al.* (2018)	ORS<br>sport drink	Sodium<br>Potassium<br>Others<br>CHO	RPE<br>Substrate utilization<br>Vo2<br>VCo2	Hct/Hb<br>Heart rate<br>Blood measures	Plasma volume changes<br>% Dehydration<br>Body weight changes<br>Sweat outputs<br>Urine outputs<br>Fluid intake<br>Respiratory loss
DS *et al.* (1991)	CHO-E polymer<br>Water	CHO<br>Sugars<br>Potassium<br>Chloride<br>Magnesium	NA	Blood measures<br>Blood measures<br>Blood cells count<br>Hct/Hb	Plasma electrolytes<br>Plasma osmolality<br>Body weight changes<br>Sweat outputs<br>Fluid intake<br>Urine outputs

Shirreffs *et al.* (2007)	gatorade<br>Water<br>Otherss	CHO<br>Sodium<br>Potassium<br>Chloride	NA	Blood measures<br>Hct/Hb<br>Subjective feelings<br>Taste perceptions	Plasma volume changes<br>Urine electrolytes<br>Plasma electrolytes<br>Sweat electrolytes<br>Body weight changes<br>Urine outputs<br>Net fluid balance<br>Plasma osmolality
Skillen *et al.* (2008)	CHO<br>A	CHO<br>Sodium<br>AA	RPE<br>Creatine kinase levels<br>Time to exhaustion<br>Muscle damage<br>Performance skill<br>Vo2<br>Respiratory ouputs	Subjective feelings<br>Blood measures<br>Hct/Hb<br>Heart rate	Body weight changes
Smith *et al.* (2017)	A-E<br>E	AA<br>Others<br>Sodium<br>Potassium<br>Calcium<br>Magnesium	Creatine kinase levels<br>Muscle damage<br>Performance skill	NA	NA
Smith *et al.* (2017)	CHO<br>A<br>CHO-A<br>Water	CHO<br>AA	Performance skill	Blood measures	NA

Snell wt al. (2010)	gatorade<br>CHO-E<br>Otherss	CHO<br>Sugars<br>Sodium<br>Calcium<br>Magnesium<br>Others	Vo2<br>Respiratory ouputs<br>Performance skill	Heart rate	Body weight changes
Matt S. *et al.* (2010)	CHO<br>CHO-A	Water<br>CHO	Muscle damage<br>Performance skill<br>Creatine kinase levels	Blood measures<br>Subjective feelings	NA
Takada *et al.* (2022)	CHO	CHO<br>Sugars<br>Sodium<br>Calcium<br>Magnesium<br>Potassium	Performance skill<br>RPE<br>Vo2<br>VCo2<br>Respiratory ouputs	Heart rate<br>Temperature<br>Hct/Hb<br>Blood measures<br>Thermal comfort	Plasma volume changes<br>Sweat outputs<br>Plasma osmolality<br>Cell volume<br>Fluid intake<br>Urine outputs<br>USG
Trong *et al.* (2015)	Otherss	AA	Performance time<br>RPE	Temperature<br>Heart rate<br>Thermal comfort	Body weight changes
Upshaw *et al.* (2016)	milk<br>CHO	CHO<br>Protein<br>Water	Performance time	Heart rate	NA
Utter AC *et al.* (2002)	CHO<br>E	Sodium<br>Potassium	RPE<br>Performance time	Blood measures	Plasma volume changes<br>Body weight changes<br>Fluid intake

Wilkerson *et al.* (2012)	Otherss	Others	Performance skill<br>Performance time<br>Power output<br>Vo2<br>PO/Vo2 ratio<br>Respiratory ouputs	Blood measures<br>Vital signs<br>Heart rate	NA
Wilson *et al.* (2016)	CHO	CHO<br>Sugars<br>Sodium<br>Chloride<br>Others	Performance skill<br>Performance time<br>Substrate utilization<br>Energy expenditure	Subjective feelings	NA
WONG *et al.* (1997)	CHO-E	Sodium<br>Potassium<br>Calcium	Vo2<br>VCo2<br>Respiratory ouputs	Subjective feelings<br>Heart rate<br>Blood measures<br>Hct/Hb	Plasma volume changes<br>Fluid intake
El-Sayed *et al.* (1996)	CHO<br>Unknown placebo	CHO	RPE<br>Vo2<br>Power output<br>Performance skill<br>Respiratory ouputs<br>Vo2<br>VCo2	Blood measures<br>Hct/Hb<br>Heart rate	Plasma volume changes<br>Body weight changes

Febbraio *et al.* (2000)	CHO-E	NR	RPE<br>Vo2<br>Performance time<br>Respiratory ouputs<br>Substrate utilization<br>VCo2	Heart rate<br>Blood measures	NA
Ferguson-Stegall *et al.* (2010)	CHO<br>CHO-PRO	CHO<br>Sugars<br>Protein	Myoglobin concentration<br>Vo2<br>RPE<br>Substrate utilization<br>Time to exhaustion<br>Performance skill<br>Respiratory ouputs<br>VCo2	Blood measures<br>Heart rate	NA
Finn *et al.* (2004)	CHO<br>Unknown placebo	NR	Total work	Blood measures<br>Subjective feelings	Body weight changes
Flood *et al.* (2020)	Water<br>CHO	Sodium Chloride<br>CHO<br>Sugars	RPE<br>Performance time<br>Total work<br>Substrate utilization<br>Respiratory ouputs<br>Vo2<br>VCo2	Hct/Hb<br>Blood measures<br>Heart rate<br>Temperature<br>Thermal comfort<br>Subjective feelings<br>Blood pH<br>Physiological strain index	Plasma volume changes

F Gilson *et al.* (2010)	milk<br>CHO	CHO<br>Protein<br>Others<br>Sodium<br>Calcium<br>Potassium<br>Others	Creatine kinase levels<br>Muscle damage<br>Myoglobin concentration<br>Performance skill	Mental fatigue<br>Subjective feelings	NA
Green *et al.* (2008)	CHO<br>CHO-PRO<br>Unknown placebo	CHO<br>Protein	Torque<br>Creatine kinase levels<br>RPE<br>Performance skill<br>Muscle damage	NA	NA
Luden *et al.* (2007)	CHO<br>CHO-PRO	CHO<br>Others<br>Protein<br>Magnesium<br>Sodium<br>Potassium	Muscle damage<br>Creatine kinase levels<br>Performance time<br>Performance skill	NA	NA
Onitsuka *et al.* (2018)	Sports drink	CHO<br>Sodium<br>Potassium	na	Temperature<br>Thermal comfort	Body weight changes<br>USG
Palmer *et al.* (1998)	CHO<br>Unknown placebo	Sodium<br>Potassium<br>CHO	Performance time<br>Power output	Heart rate	NA
Price *et al.* (2012)	unkown placebo<br>CHO-E<br>Otherss	Sodium<br>Chloride<br>CHO<br>Others	RPE<br>Power output<br>Total work<br>Respiratory ouputs	Subjective feelings<br>Heart rate<br>Blood measures<br>Blood pH	Body weight changes<br>Sweat outputs

Alan C *et al.* (2005)	CHO<br>E	Sodium<br>Potassium	RPE	NA	NA
Utter AC *et al.* (2004)	CHO<br>E	Sodium<br>Potassium	RPE<br>muscle glycogen<br>Substrate utilization<br>Vo2<br>Respiratory ouputs	Hct/Hb<br>Heart rate<br>Blood measures	Plasma volume changes
C. utter *et al.* (1999)	CHO<br>E	Sodium<br>Potassium	RPE<br>Performance skill<br>Vo2<br>Substrate utilization<br>Respiratory ouputs	Blood measures<br>Hct/Hb<br>Heart rate	Plasma volume changes
J. Valentine *et al.* (2008)	E<br>CHO<br>CHO+PRO	CHO<br>Others<br>Protein<br>Sodium<br>Potassium<br>Calcium<br>Magnesium	Creatine kinase levels<br>Myoglobin concentration<br>Muscle damage<br>Vo2<br>VCo2<br>Performance skill<br>RPE<br>Time to exhaustion<br>Respiratory ouputs	Hct/Hb<br>Blood measures<br>Heart rate	NA
Warber *et al.* (2000)	Otherss<br>Unknown placebo	NR	RPE<br>Vo2<br>Time to exhaustion	Heart rate<br>Blood measures	NA

WIDRICK *et al.* (1993)	CHO<br>Sweet placebo	CHO<br>Sugars<br>Sodium<br>Potassium	Vo2<br>VCo2<br>Substrate utilization<br>Energy expenditure<br>Power output<br>Performance time<br>mucle glycogen<br>Respiratory ouputs	Blood measures	NA
WILLIAMS *et al.* (2003)	CHO-PRO<br>Gatorade	CHO<br>Protein<br>Sodium<br>Potassium<br>Calcium<br>Magnesium<br>Others<br>Chloride	muscle glycogen	Blood measures	NA
Wojcik *et al.* (2001)	CHO<br>CHO-PRO<br>Unknown placebo	CHO<br>Protein<br>Others	Creatine kinase levels<br>muscle glycogen<br>Muscle damage<br>Performance skill<br>Torque<br>Total work	Blood measures	Urinary creatinine<br>Urine 3MH<br>Body weight changes

Legend: **Green** stand for studies reporting health outcomes, **purple** for hydration status outcomes, **blue** for performance outcomes, **pink** for hydration status and performance outcomes, **gray** for hydration status and health outcomes, **orange** for performance and health outcomes, **yellow** for hydration status, performance, and health outcomes. 3MH-I – 3-methylhistidine, AA – amino acids, ADH – antidiuretic hormone, BHI – beverage hydration index, CHO – carbohydrate, E – electrolytes, Hct/hb – hematocrit/hemoglobin concentrations, NA – non-applicable, NR – non-reported, ORS – oral rehydration solution, PCr – phosphocreatine, PO – power output, PRO – proteins, RPE – rate of perceived exertion, USG – urine specific gravity , VCo2 – volume of carbon dioxide breathe out, Vo2 – oxygen consumption.

Reporting details: No distinction between beverages in study or control beverages was made since authors were mostly not clear regarding this topic. For standardization purposes, performance outcomes were grouped into 22, health into 16, and hydration into 46 different parameters, respectively.
